# Omnichannel pricing and inventory strategies considering live streaming selling: A data-driven distributionally robust optimization approach

**DOI:** 10.1371/journal.pone.0338918

**Published:** 2026-01-07

**Authors:** Yuxia Mou, Hao Zhou, Xiaopeng Yang, Zhimin Guan

**Affiliations:** 1 School of Economics and Management, Weifang University, Weifang, China; 2 School of Business Administration, Northeastern University, Shenyang, China; 3 School of Logistics Management and Engineering, Zhuhai College of Science and Technology, Zhuhai, China; Yonsei University, KOREA, REPUBLIC OF

## Abstract

Recently, Live Streaming Selling (LSS) has become increasingly prevalent. Numerous omnichannel retailers are striving to introduce live streaming channel to absorb additional demand. However, it is challenging to investigate robust pricing and inventory strategies that consider the characteristics of omnichannel operations and LSS with uncertain demand. We consider a joint optimization of ordering, replenishment, order fulfillment, and pricing, where customers are sensitive to prices and delivery times. LSS can influence demand and benefit other channels to take free-riding. Furthermore, service level requirements are formulated as joint chance constraints to guarantee adequate performance. The Worst-case Mean Quantile-Deviation (WMQD) is employed to measure risks. The Wasserstein metric is adopted to design the data-driven ambiguity set. Accordingly, a data-driven Distributionally Robust Joint Chance Constrained Programming (DRJCCP) based on WMQD is constructed. Leveraging the dual theory, Conditional Value-at-Risk (CVaR) approximation, and linearization techniques, the developed model can be transformed into tractable formulations, which can be solved by commercial solvers. We further conduct numerical experiments to demonstrate the efficiency and practicality of our developed model. The comparative results reveal that the DRJCCP model based on WMQD has superior out-of-sample performance and is capable of effectively managing uncertainty, thereby ensuring more robust service levels. Furthermore, the sensitivity analyses are performed to verify the effects of some key parameters on the decision-making. The results indicate that introducing live streaming channel is not always profitable for the retailer and increasing the level of LSS effort can enhance free-riding effect without necessarily improving retailer’s profits.

## 1. Introduction

The global retail sector is undergoing a profound digital transformation, with Live Streaming Selling (LSS) emerging as a disruptive force. According to Coresight Research, the market size of LSS in China has reached $512 billion in 2023 (https://www.cnbc.com/2023/06/09/livestream-shopping-booms-as-small-businesses-hit-social-media-.html?&qsearchterm=livestream). The Chinese market has demonstrated unprecedented scalability for this sales model. LSS, first skyrocketed in China during the pandemic, provides real-time interaction and visual appeal. LSS can provide a more immersive and authentic experience, delivering richer product information to customers [1]. As a result, LSS demonstrates many advantages, such as reducing fitness uncertainties, enhancing customer awareness of products and alleviating customers’ psychological distance [2]. Therefore, an increasing number of omnichannel retailers have introduced live streaming channel. For example, a famous sports brand, Nike, runs its live streaming channel on Douyin in 2024 and develops a new omnichannel retailing mode (https://cn.chinadaily.com.cn/). Amazon has launched “Amazon Live Creator”, a dedicated app designed to empower its live broadcasting business (https://advertising.amazon.com/).

However, the integration of LSS into existing omnichannel frameworks poses several challenges for supply chain management, such as inventory and pricing management. Customers’ purchasing behavior during live streaming is highly uncertain and difficult to accurately predict in advance. For example, the streamer’s temporary recommendations, interactive effects, etc., may trigger impulse purchases by customers, resulting in large fluctuations in demand [2]. Orders from live streaming channel can be fulfilled from the stores apart from the DC, i.e., Ship-from-Store (SfS). In omnichannel retailing, SfS is a common implementation to fulfill online orders, which enables retailers to ship online orders from local store instead of the Distribution Center (DC). With the implementation of SfS, orders can always be delivered often cheaper and faster than from DC. However, it makes inventory management more challenging [3]. Due to the combination of online and offline demand, popular products may be sold out quickly in the physical stores, disappointing in-store customers [4]. Additionally, inventory in the DC not only supports bulk store replenishment orders but also individual customer orders. This requires the retailer to reconsider the inventory rationing and rationally allocate inventory to meet the surge in demand by the LSS. As a result, the retailer needs to develop more flexible inventory strategies to ensure adequate inventory levels, while avoiding the increased inventory costs. LSS also poses new challenges to pricing strategies. The interactive and instantaneous nature of the live streaming channel makes customers more price sensitive. As a result, the firm may use a more attractive low-pricing strategy in live streaming channel to attract more customers, while maintaining relatively high prices in other channels to maintain profit margins. However, the low-pricing strategy of live streaming channel may exert negative impacts on demands in other channels [5]. The retailer needs to coordinate pricing strategies between different channels to avoid cannibalization and ensure overall profit maximization. In addition, free-riding behavior is common in live streaming channel as its publicly accessible nature. Customer may view the live stream to obtain product introduction through live stream channel but switch to purchasing from other channels [6]. This behavior has further intensified the challenges of inventory and pricing management [7].

Furthermore, demand uncertainty is a distinctive feature in omnichannel retailing [8]. However, the challenges inherent in stochastic modeling lead many studies to exclude uncertainty analysis. Conversely, some studies assume the demand follows a well-known distribution and adopt Stochastic Programming (SP) approach [9]. Actually, the assumption of precise distribution is rarely justifiable in reality. Hence, the Robust Optimization (RO) approach is adopted in many research [10]. However, this approach considers worst-case scenarios, which can result in unnecessarily conservative decisions [11]. To alleviate the unfavorable impacts of SP and RO approaches, Distributionally Robust Optimization (DRO) is a powerful approach which assume the uncertain parameters are described by probability distributions [12]. DRO approach seeks to find an optimal solution over the worst-case probability distribution within a predetermined set, named “ambiguity set” [13]. The Wasserstein metric has attracted considerable interests from researchers, particularly because of its remarkable ability to provide finite sample guarantees [14]. Although the Wasserstein metric has been primarily utilized in modeling data-driven DRO model, no existing studies have applied it to investigate the omnichannel pricing strategies and inventory policies. Additionally, risk-averse decision-making has attracted increasing attention in the retail operations management [15]. A key research challenge is to select an appropriate risk measure that reflects the specific characteristics of the problem and its effective integration into the optimization framework. As highlighted by [16], incorporating mean-risk objective functions into optimization problems facilitates the risk measurement, such as the Mean Conditional Value-at-Risk (MCVaR) [17]. Additionally, a variety of dispersion statistics can also be employed to quantify risks. For instance, the Mean Quantile-Deviation (MQD) criterion, which exhibits convexity under specific conditions, has been proposed as a viable measure [18]. However, it is increasingly essential to focus on the Worst-case MQD (WMQD) to handle uncertainty. Nevertheless, the application of WMQD in the field of retail operations management remains limited.

Realizing these practical challenges and limitations, this paper investigates an integrated omnichannel pricing and inventory problem considering uncertain demand and service level requirement, and explore the effects of the LSS. To ensure the appropriate performance of the omnichannel network, joint chance constraints are adopted to model service level requirement. And thus, a data-driven Distributionally Robust Joint Chance-Constrained Programming (DRJCCP) based on Wasserstein metric is proposed. The aim of this work is to find a better tradeoff between the expected profit and the Quantile-Deviation (QD) under the worst-case probability distribution and determine the optimal price, ordering quantity, replenishment quantity and order fulfillment. The major contributions are shown as follows.

(1) Firstly, to the best of our understanding, this is the first paper that investigates the omnichannel retail operations incorporating LSS and service level requirements under uncertain demand, which covers a much broader scope of omnichannel retail operations than previous studies. We formulate the problem as a DRJCCP model, which integrates pricing decision with ordering, replenishment and order fulfillment decisions.
(2) Secondly, in addition to product prices in the traditional online, offline, and live streaming channels being taken into account in the demand function, the delivery time is also viewed as an important factor that influences customers’ demand. Customers have time inconsistent preferences and the quasi-hyperbolic discounting is adopted to model this behavior. Moreover, the introduction of LSS can influence demand and benefit other channels to take free-riding. Actually, this is the first study that incorporates price sensitivity, delivery time sensitivity, and free-riding into demand functions in omnichannel retailing with the LSS.
(3) Furthermore, a fundamental innovation of our work lies in the control of risks within a unified framework. Unlike prior studies, our model simultaneously addresses risks in both solution feasibility and optimality. On the one hand, we model service levels via Distributionally Robust Joint Chance Constraint (DRJCC). This ensures that all constraints are jointly satisfied with a high probability, even under the worst-case probability distribution. Additionally, we incorporate risk measure into the objective function to balance between the expected profit and the Quantile-Deviation (QD) under the worst-case probability distribution. To our knowledge, this is the first study to incorporate the joint chance constraints and risk measure into a DRO model simultaneously, which enabling a holistic approach to risk management.
(4) In addition, the Wasserstein ambiguity set is designed to depict the uncertainty of probability distribution. The data-driven DRJCCP model is transformed into a bilinear program with the Wasserstein ambiguity set, where the joint chance constraints are conservatively approximated by the worst-case CVaR constraint. By adopting piecewise affine relaxations of the bilinear terms, the data-driven DRJCCP could be further transformed into tractable formulations that could be solved directly by commercial solvers.
(5) Finally, the validity of the proposed model and the effectiveness of the solution method are evaluated by the numerical experiments. The superior performance of the WMQD-based DRJCCP is demonstrated through comparative analyses. Sensitivity analyses of fulfillment cost, technical service fee, channel preference, time-consistent preference, and free-riding are performed, which also contributes to some managerial insights for this category of problems.

The rest of this article is structured as follows. Previous relevant literature is reviewed and the significant contributions of this study are highlighted in Section 2. Section 3 describes the studied problem and formulates a stochastic chance-constrained programming model. The data-driven DRJCCP model is proposed and a tractable formulation is derived in Section 4. In Section 5, numerical experiments are conducted to validate the effectiveness of the developed model. Section 6 concludes the article and outlines future research directions. The technical proofs are included in the Appendix.

## 2. Literature review

The relevant literature is reviewed in the following three streams, i.e., omnichannel retailing, live streaming selling, and optimization methods for handling uncertainty.

### 2.1. Omnichannel retail operations management

Recently, omnichannel retail has emerged as a new standard in the retail industry, garnering significant attention from both practitioners and academics [19]. Numerous studies have focused on the omnichannel strategies. For example, Gallino and Moreno empirically investigated the impacts of Buy-Online-and-Pickup-in-Store (BOPS) on customers’ purchasing behavior and the optimal decisions [20]. Further, Gallino et al. explored whether the adoption of the Ship-to-Store (StS) can enhance the retailer’s profitability [21]. Li et al. established an analytical model to investigate the integrative strategies of ship-from-online logistics center (SFO), SfS, and StS for the retailer [22]. The aforementioned theoretical papers primarily constructed stylized models to examine the effects of omnichannel strategies on retailers’ optimal decisions and performance in simplified contexts.

The literature in omnichannel inventory management is most relevant to our study. Regarding for the inventory management, many studies investigated the joint optimization of inventory and fulfillment based on dynamics structure. For example, Govindarajan et al. proposed an inventory heuristic to determine the dynamic fulfillment and static inventory decisions [4]. Bayram and Cesaret investigated the dynamic fulfillment decisions given a fixed quantity of initial inventory [3]. Arslan et al. investigated a joint inventory and fulfillment problem for the adoption of standard shipping contracts and formulated it as a Markov decision process [23]. These studies were conducted under certain environment. However, recent literature has begun to address inventory-related issues under uncertain environment in the omnichannel retailing. Arslan et al. considered an omnichannel hierarchical decision problem and developed a two-stage SP model where the omnichannel store selection decision is made before the realization of uncertain parameters, and the inventory assignment and order fulfillment decisions can be made after observing the uncertainty [24]. Bilir further investigated the effects of the uncertain demand on the various order fulfillment strategies and inventory decisions, and the results demonstrated the importance of taking demand uncertainty into account [8]. Abouelrous et al. addressed an inventory optimization problem with stochastic demand, and the two-stage SP model was developed to approximate the problem [9]. Guo and Keskin proposed a two-stage framework, addressing inventory procurement in the first stage and order fulfillment in the second stage, and demonstrated the effects of stochastic demand structure on the SfS and BOPS strategies [25]. The abovementioned research assumed that the distributions of the uncertain parameters are known in advance. However, it is difficult to obtain the precise distributions of uncertain parameters in omnichannel retailing. Consequently, relevant studies explored omnichannel inventory-related issues based on the imprecise distribution information about uncertain parameters. Jiu explored the jointly optimization of ordering, replenishment, and fulfillment decisions in an omnichannel retailing network under demand distribution ambiguity, and a robust two-phase approach is adopted to solve the problem [26]. Lee and Moon addressed the omnichannel inventory and order fulfillment decisions when using the third-party platform as a sales channel. Similar to [26], the robust two-phase approach decoupling binary decision variables and the continuous decision variables was adopted to solve the problem [10]. Guan et al. addressed an integrated inventory control, e-fulfillment, and assortment planning problem, and proposed a DRO model based on the imprecise distribution of the uncertain parameter [27]. Sun et al. (2025) explored the omnichannel fulfillment strategies and inventory policy under the unknown distribution of uncertain demand and proposed a data-driven RO approach to deal with the uncertainties [28].

Furthermore, many scholars incorporate pricing decisions into the omnichannel inventory management problems. Actually, in many studies, price is an important factor that influences customers’ purchasing decisions. For example, Gupta et al. addressed the inventory control and price optimization problems for the omnichannel retailers and modeled demand function using mixed logit model incorporating prices [29]. Kusuda investigated the optimal inventory control and pricing decisions and obtained two types of rational expectation equilibrium: with and without BOPS [30]. Additionally, some literature addressed the pricing and inventory problem under uncertain environment, such as [31,32]. Qiu et al. proposed a robust pricing and inventory optimization model that considered different return policies with demand uncertainty [31]. Sun et al. addressed the pricing and inventory decisions with the SfS strategy and developed a RO model under a budgeted uncertainty set [32].

Various studies have considered the omnichannel retail operations management from the perspective of pricing, inventory, and order fulfillment decisions. However, live streaming selling has rapidly emerged as an innovative, highly interactive sales model, profoundly altering customers’ purchasing behavior and decision-making. The unique characteristics of this channel present challenges and opportunities for omnichannel retail operations management, yet existing research has not adequately addressed these developments.

### 2.2. Live streaming selling (LSS)

The increasing popularity of the LSS has attracted attention from both academics and practitioners. He et al. [33] and Zhang et al. [34] have relied on empirical approaches to examine what motivations in live streaming can drive customers to engage and purchase in live streaming channel, such as customers’ trust, the emotional requirement for identification, informative and persuasive effect, interactivity effect. In addition, many studies investigated the impacts of LSS on the operational performance and explored how the supply chain members can benefit from LSS by adjusting their operational strategies. The preceding studies developed analytical models to address pricing problem. For instance, Lin et al. examined a trade-off between price negotiation and profit margins based on a Nash bargaining game considering the LSS [35]. Chen et al. proposed a two-stage model to investigate how the live streaming selling interacts with different pricing strategies [36]. Ku et al. developed an analytical model by considering the differentiated pricing, customer segmentation, and demand expansion derived from LSS, and demonstrated that LSS was not always beneficial for the e-retailer [37].

Recent research, building on the pricing decisions, has shifted towards exploring optimal channel strategies related to LSS. Gong et al. investigated the live streaming strategy under multichannel sales modes for an online retailer, and found that the effects of LSS relied on the product standardization and quality [38]. Pan et al. pointed out that adopting LSS is profitable only when the influencers’ selling level is significant high [39]. Huang et al. examined the effects of live streaming channel introduction considering competing retailers, and demonstrated that the LSS might not always enhance the introducers’ demand or benefit the retailer with free-riding customers [1]. Zhou et al. developed a duopoly competition game model to explore the optimal live streaming channel strategy. The results indicated that the decision to choose between the human-hosted and AI-supported live streaming hinged on the hassle costs [40].

Notably, the literature mentioned above assumes that the traditional online channel already exists, and further explores the live streaming channel strategies as well as pricing problem. However, as an emerging practice in omnichannel retailing, LSS also exerts nonnegligible impacts on the offline channel. In our study, we try to simultaneously explore the channel introduction strategies and examine the impacts of the LSS on traditional online and offline channels. In addition, unlike the above literature that adopts empirical studies or game-theoretic models, our research uses a data-driven DRJCCP model to investigate the live streaming channel operations problem with uncertainty demand.

### 2.3. Optimization approaches for handling uncertainty

The SP and RO are two commonly used method to deal with uncertainty in the optimization field. In the case of SP, the probability distributions of the uncertain parameter are assumed to be exactly available in advance [41]. The problem tends to identify the decision variables that either maximizes or minimizes the expected profits or costs based on the specified probability distributions. However, in real world applications, it is challenging to estimate the accurate probability distribution of the uncertain parameter [42]. In the case of RO approach, it is assumed that the uncertain parameter belongs to a specified ambiguity set, and only optimize the objective function over the worst-case bound, which ensures that the optimal solution is feasible for all potential scenarios. Hence, the RO is the most conservative approach [43]. The DRO approach, generalizing the SP and RO, can compensate for the inherent drawbacks of SP and RO [44]. The DRO approach assumes the exact probability distribution for the uncertain parameter remains unknown, and identifies the optimal solutions by considering the worst-case distribution within an ambiguity set containing the true distribution [45]. The problem will become the SP when the candidate distribution in the ambiguity set contains only the true distribution. On the other hand, the problem will become the RO when the problem considers all distributions under the given support set. It offers solutions are less conservative than those provided by traditional RO approach while maintaining the computational tractability inherent in the primal deterministic formulation [46,47].

Additionally, the performance of the DRO model is greatly dependent on the ambiguity sets [48]. Recently, the Wasserstein-metric-based DRO approach has been widely adopted in the field of operations management. For example, Saif and Delage explored the classical capacitated facility location problem using the DRO model based on Wasserstein-metric-based ambiguity set [49]. Shehadeh studied a stochastic surgery planning problem under the uncertain durations of elective and emergency surgeries and proposed a DRO model with the Wasserstein-metric-based ambiguity set to address distributional uncertainty [50]. Kim and Chung developed the DRO model with the Wasserstein-metric-based ambiguity set and investigated the benefit of the dual-sourcing strategy in inventory management [13]. Liu et al. proposed a two-stage DRO model to address healthcare resource pre-positioning and patient scheduling problem and introduced a Wasserstein distance-based ambiguity set to deal with the limited data challenge [51]. However, there is very little literature using the Wasserstein-metric-based DRO approach to handle uncertainty in omnichannel retailing management. Although Momen and Torabi used the Wasserstein-metric-based DRO approach in the omnichannel system, they examined a dynamic competition through a Nash-Stackelberg game framework [52]. Furthermore, under the Wasserstein metric, the distributionally robust chance constraint (DRCC) has been studied in supply chain and logistics related fields. For example, Hashemi-Amiri et al. addressed a joint optimization problem of supplier selection, production scheduling, and vehicle routing problem, and DRCC approach with the Wasserstein metric was applied to ensure the demand and capacity are satisfied with high probability [53]. Niu et al. explored a capacity-sharing supply chain network optimization problem and formulated a DRCC based on the Wasserstein metric to address uncertainties [54]. Wang et al. developed an ambiguous joint chance constraint with the Wasserstein metric to study the hazardous products supply chain design problem [55]. However, there is no literature on the adoption of DRCC in omnichannel retailing related studies.

Under the uncertain environment, risk-neutral methods exhibit the potential drawbacks, namely, extremely pessimistic worst-case solutions or the adverse impacts of excessive variability in decisions. Therefore, it is imperative to consider risk aversion with the uncertainty [56]. Actually, risks can be addressed by adopting virous risk measurement tools, such as Conditional Value-at-Risk (CVaR) [57], which is a coherent risk measure. However, Ahmed has pointed out that it is beneficial to consider the mean-risk objection function for measuring risks [16]. For example, Guan et al. adopted the MCVaR criterion to measure the risks in a capital-constrained fresh product supply chain [17]. Additional, dispersion statistics can also be employed to quantify risks, such as the MQD criterion, while the conventional MQD lacks robustness for handling uncertainty. To address this shortcoming, it is increasingly essential to focus on the Worst-case MQD (WMQD). For example, Wang et al. [18] proposed a risk-averse DRO model for the disaster relief logistics problem by using the WMQD criterion, and proved that this measure can avoid the conservatism of the Worst-case MCVaR (WMCVaR) criterion. In our study, the WMQD criterion will be adopted to measure risks.

### 2.4. Research gaps and contributions

We briefly review the most relative studies to identify the research gaps. As presented in Table 1, firstly, there are very few papers that integrate pricing, inventory, and order fulfillment decisions with uncertainty. Moreover, there is a scarcity of research that addresses the integration of LSS within the domain of omnichannel retail operations. We aim to concurrently investigate the channel introduction strategies and examine the impacts of the LSS on traditional online and offline channels. Furthermore, a data-driven DRJCCP model based on WMQD has not been adopted in the study of omnichannel retailing. Therefore, we address an integrated pricing, inventory, order fulfillment optimization problem with demand uncertainty and service level requirement, and explore the effects of the introduction of live streaming channel. A data-driven DRJCCP is developed based on Wasserstein metric, and WMQD criterion is adopted to find a better tradeoff between the expected profit and the risks under the worst-case probability distribution.

**Table 1:** A summary of relative literature.

Reference	Decisions			Service level	LSS	Risk measure^a^	Data-driven	Modelling method
	Pricing	Inventory	Fulfillment					
Arslan et al. [24]		√	√			None		SP
Abouelrous et al. [9]		√	√			None		SP
Bayram & Cesaret [3]			√			None		Deterministic
Momen & Torabi [52]	√	√	√			None	√	DRO
Govindarajan et al. [4]		√	√			None		Deterministic
Arslan et al. [23]		√	√			None		Deterministic
Jiu [26]		√	√			None		RO
Bilir [8]		√	√	√		None		SP
Lee & Moon [10]		√	√			None		RO
Qiu et al [31]	√	√				None	√	RO
Sun et al. [32]	√	√	√			None	√	RO
Guan et al. [27]		√	√			WMCVaR		DRO
This work	√	√	√	√	√	WMQD		DRJCCP

^a^Risk measure: Conditional Value-at-Risk (CVaR), Worst-case Conditional Value-at-Risk (WCVaR), Worst-case mean Conditional Value-at-Risk (WMCVaR), Worst-case mean Quantile-Deviation (WMQD).

## 3. Problem description and mathematical model

### 3.1. Problem description

**Fig 1:**
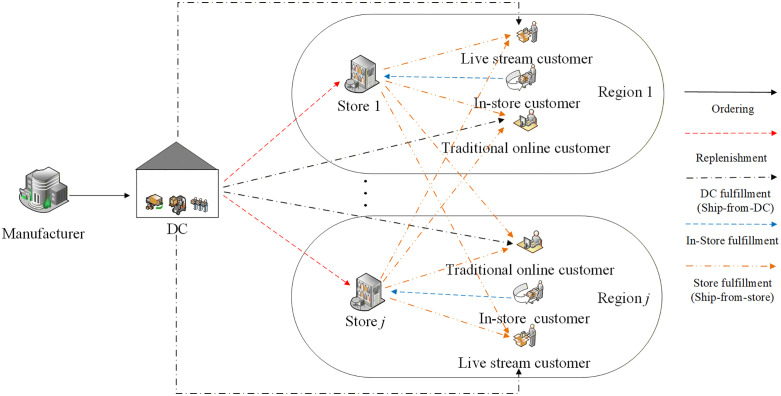
The omnichannel retail network.

In this study, a single period, single product, and multi region omnichannel retail network is considered. As depicted in Fig 1, the omnichannel retailer, hereinafter referred to as retailer, planning for selling the seasonal product to customers, orders products from the manufacturer, and replenishes products from the DC to stores. The DC and stores are operated by the retailer. We assume the store j is located in region j, and there is only a single store within each region. For the sake of simplicity, we just show two regions in Fig 1. Nonetheless, in the problem we study, the retailer has several such regions.

The retailer has set up stores in several regions and already have a well-established omnichannel retailing system. However, with the rapid development of LSS, many retailers have cooperated with live streaming platforms to selling products. Therefore, we consider the retailer intends to add live streaming channel into its operations to capture a larger market share. The retailer opts for the self-broadcast sales strategy where the retailer need to set up their own live streaming studios in the live streaming platform and establish their own live streaming teams. Customers can purchase products from three types of channels: (i) the offline channel ***f***; (ii) the traditional e-channel ***o***, also known as the traditional online channel; and (iii) the live streaming channel ***b***. Accordingly, the offline channel demand in any given region is satisfied through the specified store in this region. The traditional e-channel demand and live streaming order are fulfilled through omnichannel delivery. Omnichannel delivery refers to the integration of multiple strategies to provide a seamless and consistent customer experience. Specifically, in our study, when the traditional e-channel and live streaming orders arrive, the retailer can choose to fulfill these orders either from the DC or store (i.e., SfS). When the specified store is out-of-stock and cannot fulfill these online orders, store clerks can check the availability of products in other stores through the inventory sharing system, and designate these orders to other nearby stores. This approach ensures that customers receive their orders in the most efficient and convenient manner. The difference between these two types of demands is that the delivery time for live streaming orders is longer than that for traditional e-channel orders. Live streaming orders tend to arrive more centrally, whereas traditional online orders arrive in a sequence. As a result, the time taken to process live streaming orders is longer than online orders. Furthermore, to guarantee adequate performance of omnichannel operations, the retailer requires to ensure that certain service levels are satisfied in all channels.

Some important assumptions are made below.

**Assumption 1.** The prices of the same product in different regions are equal in online and offline channels, but the prices in live streaming channel are often lower than them. This is common in real practice because product prices in live streaming channel are often discounted [35]. For example, Adidas, as one of the sports brand giants, often conducts intense discount promotions during the LSS to boost sales.

**Assumption 2.** Price and delivery time are non-negligible factors that affect customer demand. An increase in price often leads to a decrease in demand, and a portion of customers may switch to other channels because of price differences between channels. In addition, long delivery time often results in demand decreasing in traditional e-channel and live streaming channel [58].

**Assumption 3.** There exists a priority for inventory allocation in stores: offline channel>traditional e-channel>live streaming channel. In general, the demand in offline channel is viewed as the first priority. If the desired product is out of stock in the store when customers go to store and intend to make purchase, it will seriously affect the retailer’s reputation, and result in the loss of customers [59]. Haddon observed that “when walk-in customers do not immediately find the items they want, 70% of customers will switch to another store or brand” [60]. In addition, Uniqlo, as a retail industry practice, also illustrates this phenomenon. Uniqlo will remove products from its online channel when in-store inventory falls below a certain threshold, to ensure that stores have sufficient inventory to promptly serve walk-in customers and to prevent conflicts between in-store and online demands. Demand in live streaming channel may be given the lowest priority. Live streaming order arrivals tend to be concentrated, with orders typically being sent out after the live streaming has ended. However, traditional e-channel orders are often dispersed and typically arrive in a sequence. Primary drivers for live-streaming shoppers are always price promotion and interactive entertainment. Consequently, these customers generally have lower inherent expectations for delivery time compared to traditional online customers [61].

**Assumption 4.** Fulfillment costs for traditional online and live streaming channels are related to delivery times. The fulfillment costs can be calculated by $m^{ful}=\underset{―}{m}+a/L$, where $\underset{―}{m}$ represents the minimum fulfillment cost, a signifies the marginal increase in the fulfillment cost, and *L* represents the delivery time [29].

**Assumption 5.** The live streaming platform will charge the technical service fee (ε), also known as commission rate, from the retailer when customers purchase products from live streaming room. In practice, technical service fee is determined exogenously before the retailer entries the live streaming platform. Empirical data indicates that ε varies from 5% to 12% on JD.com, while on Tiktok, ε is changed from 2% to 5% for most of the regular categories and it is 10% when the sales reach or exceed 5,000¥ [62]. In addition, ε on Amazon.com generally falls within a range of 5% to 45%, although it may be higher for some special categories [63]. Therefore, according to [2], ε is assumed to fall within the range of [5%, 50%].

**Assumption 6.** Assuming the costs of live streaming, including venue and equipment costs, revenue sharing with the streamers, etc., $nv^{2}$, where n represents the unit cost of LSS efforts, and v denotes the level of LSS efforts. The quadratic cost function is proposed to capture the fact that costs increase as the level of LSS efforts increases, but marginal returns decrease. This follows the assumption that the rational decision-maker always targets the “lowest-hanging fruit”, leading to subsequent enhancement becoming more difficult [62]. Moreover, the level of LSS efforts v is treated as an exogenous, pre-determined parameter rather than an endogenous decision variable. This modeling choice is made to isolate and focus on the subsequent operational decisions, specifically pricing and inventory management, and to analyze their optimal responses to a given marketing intensity.

In summary, we address a joint optimization problem of pricing, ordering, replenishment and order fulfillment over a single selling season with LSS in omnichannel retailing system. The key events of the selling season can be characterized as occurring before, during, or after the selling season. Specifically, before the selling season, the retailer first makes pricing decision for each channel. And then, based on market forecasts, the retailer decides on the ordering quantity from the manufacturer. After storing the product in the DC, the retailer need to decide how much to replenish the store inventory. Additionally, the retailer makes the order fulfillment decision that chooses from which stores or DC to retrieve the products to fulfill traditional online or live streaming demand. After the selling season, the retailer will dispose of the remaining product; or the retailer requires to pay the shortage cost if customer demand is not satisfied.

The following notations in Table 2 are adopted to establish the model.

**Table 2:** The list of notations.

**Indexes**	
j	Existing DC and store (region) index, $j\in J^{\prime }=J\cup \{0\}$, where J={1,2,...} is the set of stores (regions) and 0 represents the DC
***g***	Channel index, g∈G={f,o,b}, where ***f***, ***o***, ***b*** represent the offline channel, tradition e-channel, and live streaming channel, respectively
**Input parameters**	
w	Unit ordering cost of the retailer
$c_{j}$	Unit replenishment cost for the store in region j
$m_{j^{\prime }j}^{o}$, $m_{j^{\prime }j}^{b}$	Unit order fulfillment cost for the demand in channel **o** and ***b***, respectively, from DC or store $j^{\prime }$ to customers in region j
${\underset{―}{m}}_{j^{\prime }j}^{o}$, ${\underset{―}{m}}_{j^{\prime }j}^{b}$	Minimum unit fulfillment cost for the demand in channel ***o*** and ***b***, respectively, from DC or store $j^{\prime }$ to customers in region j
a	Marginal increase in the fulfillment cost
$s_{j}^{g}$	Unit shortage cost of channel ***g*** in region j
$h_{j}$	Unit inventory holding cost of product in DC or store j
n	Unit cost of LSS efforts
$\xi_{j}$	Potential market size in region j, which is a stochastic variable
$\theta_{j}^{g}$	Customer channel preference ratio toward channel ***g*** in region j
$\alpha_{j}^{g}$	Self-price elasticity in channel ***g*** in region j, $\text{0}<\alpha_{j}^{g}<\text{1}$
$\beta_{j}$	Cross-price elasticity for customers in region j, $0<\beta_{j}<1$
e, γ	Short-term factor and long-term factor, respectively
$L^{o}$, $L^{b}$	Delivery time in channel ***o*** and ***b***, respectively
$\lambda_{j}$	Customers’ sensitivity towards streamer’s selling efforts in region j
ε	Technical service fee
$\omega_{j}^{o}$, $\omega_{j}^{f}$	The degree of free-riding in channel ***o*** and ***f*** in region j, where $\text{0}<\omega_{j}^{o},\omega_{j}^{f}<\text{1}$, and let $\omega_{j}^{b}=\text{1}-\omega_{j}^{o}-\omega_{j}^{f}$.
$\kappa^{g}$	Chance constraint parameter of channel ***g***, $\text{0}<\kappa^{g}<\text{1}$
v	Level of LSS efforts
**Decision variables**	
$p_{j}^{g}$	Retail price in channel ***g*** in region j, where $p_{j}^{f}=p_{j}^{o}=p_{j}>p_{j}^{b}$
Q	Ordering quantity from manufacturer
$I_{j}$	Replenishment quantity from DC to store j
$S_{j^{\prime }j}^{o}$, $S_{j^{\prime }j}^{b}$	Quantity of demand in region j fulfilled by DC or store $j^{\prime }$ in channel ***o*** and ***b***, respectively
$S_{j}^{f}$	Quantity of in-store demand satisfied in region j

### 3.2. Mathematical model

#### 3.2.1. The objective function.

A popular approach to measuring risks is to construct a weighted objective on expectations and risks. Actually, various discrete statistics can be adopted to measure risks [16]. A new mean-risk objective function, namely, WMQD, is adopted to measure risks. Additionally, we would compare WMQD with another popular risk measure, i.e., WMCVaR. This subsection provides brief definitions of WMCVaR and WMQD in the context of profit maximization. Firstly, according to [64], the definitions of VaR and CVaR are given as follows.

**Definition 1.** Let f(x,y) be the profit function associated with the decision vector x, to be chosen from the subset X of ${\mathbb{R}}^{n}$. $y\in {\mathbb{R}}^{m}$ represents the stochastic parameter, where p(y) is the probability density function of y. Then $\Phi (x,\delta )=\int_{f(x,y)\geq \delta }p(y)dy$ can be used to represent the probability that f(x,y) is not less than the threshold δ, where the distribution function related to x is denoted by Φ(x,δ). $VaR_{\tau }=max\{\delta \in {\mathbb{R}}^{+}|\Phi (x,\delta )\geq 1-\tau \}$, which is denoted as the threshold at which the probability that the profit function exceeds δ is no less than 1−τ. Note that, τ∈[0,1] denotes the risk-aversion degree for decision- maker, the smaller τ is, the more risk-averse the decision-maker is.

**Definition 2.** CVaR measures average profit falling below the quantile level set by VaR, which is denoted by $CVaR_{\tau }\left(f\left(x,y\right)\right)=\frac{\text{1}}{\tau }\int_{f(x,y)\leq VaR_{\tau }}f(x,y)p(y)dy$. Further, Rockafellar and Uryasev proved that CVaR has an equivalent definition as $CVaR_{\tau }\left(f\left(x,y\right)\right)=\max F_{\tau }\left(x,\delta \right)$, where $F_{\tau }\left(x,\delta \right)=\delta -\frac{\text{1}}{\tau }𝔼{\left[\delta -f\left(x,y\right)\right]}^{+}$, ${\left[\partial \right]}^{+}=\max\left\{\partial ,0\right\}$, and 𝔼[·] denotes the expectation value operator [64].

CVaR, which is defined based on VaR, has many superior properties. It is a coherent risk measurement tool, as it follows convexity, monotonicity, translation equivariance and positive homogeneity [64].

$QD_{\tau }\left(f\left(x,y\right)\right)$, denoting the quantile-deviation for a stochastic variable x at confidence level τ, is defined as


$$QD_{\tau }\left(f\left(x,y\right)\right)=\min\left\{𝔼\left[\left(1-\tau \right){\left(VaR_{\tau }-f\left(x,y\right)\right)}^{+}+\tau {\left(f\left(x,y\right)-VaR_{\tau }\right)}^{+}\right]\right\},$$ (1)

where 𝔼[·] represents the expect value operator, and ${\left(\partial \right)}^{+}=\max\left\{\partial ,0\right\}$. According to [66], $CVaR_{\tau }\left(f\left(x,y\right)\right)$ can be expressed by $QD_{\tau }\left(f\left(x,y\right)\right)$ as follows:


$$CVaR_{\tau }\left(f\left(x,y\right)\right)=𝔼\left(f\left(x,y\right)\right)-\frac{\text{1}}{\tau }QD_{\tau }\left(f\left(x,y\right)\right).$$ (2)

Both QD and CVaR can overcome the limitation of VaR, and they have better computational properties: convexity, monotonicity, translation equivariance and positive homogeneity, among others [64–66].

According to [16,65], the mean QD objective


$$MQD_{\tau }\left(f\left(x,y\right)\right)=𝔼\left(f\left(x,y\right)\right)+\chi QD_{\tau }\left(f\left(x,y\right)\right),$$ (3)

is concavity preserving for all χ∈[0,1/1(1−τ)\nulldelimiterspace(1−τ)].

The mean CVaR objective can be formulated as


$$MCVaR_{\tau }\left(f\left(x,y\right)\right)=𝔼\left(f\left(x,y\right)\right)+\chi CVaR_{\tau }\left(f\left(x,y\right)\right),$$ (4)

is concavity preserving for all χ≥0.

Non-negative weight χ can weights expected profit against risk. The decision-maker is risk-neutral and only focuses on revenue when χ=0. The decision-maker can easily move from risk-neutral to risk-averse by increasing the value of χ.

The WMQD and WMCVaR, which represent the MQD and MCVaR under the worst case, can be defined as $WMQD\left(f(x,y)\right)=\inf_{p(y)\in 𝒟}\;\;MQD\left(f(x,y)\right)$ and $WMCVaR\left(f\left(x,y\right)\right)=\inf_{p(\cdot )\in 𝒟}\;\;MCVaR\left(f\left(x,y\right)\right)$, respectively, where inf(·) represents the worst-case scenario, and we assume the density function is only known to belong to a certain set 𝒟.

The retailer’s total profit across all channels can be formulated as:


$$\begin{array}{cc} ℋ= & \left[\sum_{j\in J}p_{j}\left(S_{j}^{f}+\sum_{j^{\prime }\in J^{\prime }}S_{j^{\prime }j}^{o}\right)+(1-\varepsilon )\sum_{j\in J}\sum_{j^{\prime }\in J^{\prime }}p_{j}^{b}S_{j^{\prime }j}^{b}\right]-[h_{\text{0}}{\left(Q-\sum_{j\in J}I_{j}-\sum_{g\in G\backslash \{f\}}\sum_{j\in J}S_{\text{0}j}^{g}\right)}^{+}\;\\ & -\;\sum_{j\in J}h_{j}{\left(I_{j}-S_{j}^{f}-\sum_{g\in G\backslash \{f\}}\sum_{j^{\prime }\in J}S_{jj^{\prime }}^{g}\right)}^{+}]-wQ-\sum_{j\in J}c_{j}I_{j}-\sum_{g\in G\backslash \{f\}}\sum_{j\in J}\sum_{j^{\prime }\in J^{\prime }}m_{j^{\prime }j}^{g}S_{j^{\prime }j}^{g}\\ & -\left[{\sum_{j\in J}s_{j}^{f}\left({\hat{D}}_{j}^{f}-S_{j}^{f}\right)}^{+}+\sum_{g\in G\backslash \{f\;\}}\sum_{j\in J}s_{j}^{g}{\left({\hat{D}}_{j}^{g}-\sum_{j^{\prime }\in J^{\prime }}S_{j^{\prime }j}^{g}\right)}^{+}\right]-nv^{\text{2}}. \end{array}$$ (5)

The first term in Eq. (5) is the total revenue of offline, traditional online and live streaming channels. The second term represents the inventory holding costs of remaining product in the DC and stores. The third and fourth terms are the ordering costs and replenishment costs, respectively. The next term denotes the total order fulfillment costs in traditional online and live streaming channels. The sixth term represents the shortage costs of offline, traditional online and live streaming channels. The last term is the costs of LSS efforts. For notational convenience, let boldface glyghs, $\xi ={\left(\xi_{\text{1}},\xi_{\text{2}},...,\xi_{\left|J\right|}\right)}^{\text{T}}$, $p={\left(p_{\text{1}},p_{\text{2}},...,p_{\left|J\right|}\right)}^{\text{T}}$, $p^{b}={\left(p_{\text{1}}^{b},p_{\text{2}}^{b},...,p_{\left|J\right|}^{b}\right)}^{\text{T}}$, $\lambda ={\left(\lambda_{\text{1}},\lambda_{\text{2}},...,\lambda_{\left|J\right|}\right)}^{\text{T}}$, $S^{f}={\left(S_{\text{1}}^{f},S_{\text{2}}^{f},...,S_{\left|J\right|}^{f}\right)}^{\text{T}}$, $h={\left(h_{\text{1}},h_{\text{2}},...,h_{\left|J\right|}\right)}^{\text{T}}$, $c={\left(c_{\text{1}},c_{\text{2}},...,c_{\left|J\right|}\right)}^{\text{T}}$, $I={\left(I_{\text{1}},I_{\text{2}},...,I_{\left|J\right|}\right)}^{\text{T}}$, $m_{j}^{o}={\left(m_{\text{0}j}^{o},m_{\text{1}j}^{o},...,m_{\left|J^{\prime }\right|j}^{o}\right)}^{\text{T}}$, $m_{j}^{b}={\left(m_{\text{0}j}^{b},m_{\text{1}j}^{b},...,m_{\left|J^{\prime }\right|j}^{b}\right)}^{\text{T}}$, $s^{g}={\left(s_{\text{1}}^{g},s_{\text{2}}^{g},...,s_{\left|J\right|}^{g}\right)}^{\text{T}}$, denote the vectors with $\xi_{j}$, $p_{j}$, $p_{j}^{b}$, $L_{j}^{o}$, $L_{j}^{b}$, λ, $S_{j}^{f}$, $h_{j}$, $c_{j}$, $I_{j}$, $m_{j^{\prime }j}^{o}$, $m_{j^{\prime }j}^{b}$, $s_{j}^{g}$, as the *j*-th element in the respective vectors. Let $S^{𝐨}={\left(\sum {\mathrm{\backslash nolimits}}_{j^{\prime }\in J^{\prime }}S_{j^{\prime }\text{1}}^{o},\sum {\mathrm{\backslash nolimits}}_{j^{\prime }\in J^{\prime }}S_{j^{\prime }\text{2}}^{o},...,\sum {\mathrm{\backslash nolimits}}_{j^{\prime }\in J^{\prime }}S_{j^{\prime }|J|}^{o}\right)}^{\text{T}}$ and $S^{b}={\left(\sum {\mathrm{\backslash nolimits}}_{j^{\prime }\in J^{\prime }}S_{j^{\prime }\text{1}}^{b},\sum {\mathrm{\backslash nolimits}}_{j^{\prime }\in J^{\prime }}S_{j^{\prime }\text{2}}^{b},...,\sum {\mathrm{\backslash nolimits}}_{j^{\prime }\in J^{\prime }}S_{j^{\prime }|J|}^{b}\right)}^{\text{T}}$, where $S_{j}^{o}={\left(S_{0j}^{o},S_{1j}^{o},...,S_{\left|J^{\prime }\right|j}^{o}\right)}^{T}$, $S_{j}^{b}={\left(S_{\text{0}j}^{b},S_{\text{1}j}^{b},...,S_{\left|J^{\prime }\right|j}^{b}\right)}^{\text{T}}$, $S_{\text{0}}^{o}={\left(S_{\text{0}\text{1}}^{o},S_{\text{0}\text{2}}^{o},...,S_{\text{0}|J|}^{o}\right)}^{\text{T}}$, $S_{\text{0}}^{b}={\left(S_{\text{0}\text{1}}^{b},S_{\text{0}\text{2}}^{b},...,S_{\text{0}|J|}^{b}\right)}^{\text{T}}$. Let $\Theta^{f}$, $\Theta^{o}$, $\Theta^{b}$, ${𝐀}^{g}$, **B**, $\Omega^{g}$ denote the |J|×|J| dimensional diagonal matrixes with $\theta_{j}^{f}$, $e\gamma^{L^{o}}\theta_{j}^{o}$, $e\gamma^{L^{b}}\theta_{j}^{b}$, $\alpha_{j}^{g}$, $\beta_{j}$, $\omega_{j}^{g}$ as elements j on the main diagonal of the respective matrixes. $\theta_{j}^{f}$, $e\gamma^{L^{o}}\theta_{j}^{o}$, $e\gamma^{L^{b}}\theta_{j}^{b}$, $\alpha_{j}^{g}$, $\beta_{j}$, $\omega_{j}^{g}$ are columns j of the matrixes $\Theta^{f}$, $\Theta^{o}$, $\Theta^{b}$, ${𝐀}^{g}$, **B**, $\Omega^{g}$. **1** is a vector of appropriate dimensions, where all elements are equal to 1. The auxiliary variables/vectors $\sigma_{0}$, $\;\sigma ={\left(\sigma_{\text{1}},\sigma_{\text{2}},...,\sigma_{\left|J\right|}\right)}^{\text{T}}$ and $\;\varphi^{g}={\left(\varphi_{\text{1}}^{g},\varphi_{\text{2}}^{g},...,\varphi_{\left|J\right|}^{g}\right)}^{\text{T}}$ are introduced to replace the remaining inventory in the DC and stores, and unsatisfied demand of each channel, respectively. Therefore, the vector-based profit function is denoted as:


$$\begin{array}{cc} \;ℋ=\; & p^{\text{T}}\left(S^{f}+S^{o}\right)+(\text{1}-\varepsilon ){\left(p^{b}\right)}^{\text{T}}S^{b}-h_{\text{0}}\sigma_{\text{0}}-h^{\text{T}}\sigma -wQ-c^{\text{T}}I-\sum_{j\in J}{\left(m_{j}^{o}\right)}^{\text{T}}S_{j}^{o}\\ & \;\;-\sum_{j\in J}{\left(m_{j}^{b}\right)}^{\text{T}}S_{j}^{b}-{\left(s^{f}\right)}^{\text{T}}\varphi^{f}-{\left(s^{o}\right)}^{\text{T}}\varphi^{o}-{\left(s^{b}\right)}^{\text{T}}\varphi^{b}-nv^{\text{2}}. \end{array}$$ (6)

Thus, the $\;{\text{MQD}}_{\tau }\left(ℋ\right)$ can be expressed as follows:


$$\;{\text{MQD}}_{\tau }\left(ℋ\right)=E\left(ℋ\right)+\chi {\text{QD}}_{\tau }\left(ℋ\right).$$ (7)

#### 3.2.2. The demand function.

According to [31], the demand in different channels can be characterized by linear functions. Customer demands in each channel are uncertain due to business-as-usual factors and catastrophic events. The demands in offline, traditional online and live streaming channels, denoted by $\;{\hat{D}}_{j}^{f}$, $\;{\hat{D}}_{j}^{o}$ and $\;{\hat{D}}_{j}^{b}$, respectively, are expressed by


$$\;{\hat{D}}_{j}^{f}=\theta_{j}^{f}\xi_{j}-\alpha_{j}^{f}p_{j}^{f}+\beta_{j}p_{j}^{b}+\omega_{j}^{f}\lambda_{j}v,\forall j\in J,$$ (8)

$$\;{\hat{D}}_{j}^{o}=e\gamma^{L^{o}}\theta_{j}^{o}\xi_{j}-\alpha_{j}^{𝐨}p_{j}^{o}+\beta_{j}p_{j}^{b}+\omega_{j}^{o}\lambda_{j}v,\forall j\in J,$$ (9)

$$\;{\hat{D}}_{j}^{b}=e\gamma^{L^{b}}\theta_{j}^{b}\xi_{j}-\alpha_{j}^{b}p_{j}^{b}+\beta_{j}p_{j}+\omega_{j}^{b}\lambda_{j}v,\forall j\in J,$$ (10)

where $\xi_{j}$ is uncertain parameter defined in the intervals of $\;[{\underset{―}{\xi }}_{j},{\overset{―}{\xi }}_{j}]$. Noted that $\;\sum_{g\in G}\theta_{j}^{g}=\text{1}$ and $\;\text{0}<\theta_{j}^{g}<\text{1}$, for region j. $\;\alpha_{j}^{f}$, $\;\alpha_{j}^{o}$ and $\;\alpha_{j}^{b}$ are the self-price elasticities of customers in region j for offline, traditional online and live streaming channels, respectively. Additionally, $0<\beta_{j}<1$ is the cross-price elasticity of customers in region j. To keep analysis tractable, we assume the cross-price elasticity is symmetric. Additionally, $\;\alpha_{j}^{f}>\beta_{j}$, indicating that customers are less sensitive to the retail price in the competitive channels.

Furthermore, customers are sensitive to the delivery time and reveal time-inconsistent preferences. Quasi-hyperbolic discounting is adopted to model this behavior. Under classic exponential discounting, it is assumed that customers have perfectly rational expectations regarding future costs and benefits, and that their preferences remain stable over time. However, experimental evidence has revealed that this model does not accurately reflect how customers truly perceive the value of time; they tend to place greater emphasis on the near future compared to the distant future [67]. To better capture these experimental findings, Loewenstein and Prelec introduced hyperbolic discounting to represent time inconsistency, while its inherent complexity often causes the intractability of theoretical analysis [67]. Therefore, Laibson introduced the quasi-hyperbolic discounting, which can capture the essence of hyperbolic discounting while overcome analytical intractability [68]. Under this function, all the payments and payoffs occurring in the current period are undiscounted, while those occurring in the future are discounted. Empirical studies have shown that the quasi-hyperbolic discounting closely aligns with experimental data [69], and it successfully explains the deviation from consistent preferences and biases in customer’s decisions observed in field studies [70]. In addition, this function has been widely adopted in retail research, as evidenced by studies such as [71,72]. In our study, the initial demands in traditional online and live streaming channels are discounted by $\;e\gamma^{L^{o}}$ and $\;e\gamma^{L^{b}}$, respectively. Hyperbolic discounting consists of two different systems of discounting. One is the standard exponential discounting, in which demands satisfied at delivery time in traditional online and live streaming channels are discounted by $\;\gamma^{L^{o}}$ and $\;\gamma^{L^{b}}$, respectively, where γ∈[0,1] is the long term factor. The other applies to all future demand regardless of the interval of delay; the demand without instant satisfaction is discounted by e, and e∈[0,1] is the short-term factor.

Moreover, LSS can influence customers’ purchase decision. Clearly, the higher level of LSS efforts will create a more sales volume. The expression of $\lambda_{j}v$ indicates additional demand due to retailer’s LSS efforts. However, some customers may prefer to learn about the product immersively in live streaming rooms, but turn to traditional online or offline channels to purchase products. In fact, the retailer’s selling efforts in live streaming channel can benefit for other channels to take free-riding. Namely, $\;\omega_{j}^{f}\lambda_{j}v$ and $\;\omega_{j}^{o}\lambda_{j}v$ represent the positive spillover effects of LSS on offline and traditional online channels, respectively, and $\;\omega_{j}^{b}\lambda_{j}v$ denotes the increased demand in live streaming channel due to LSS, where $\;\omega_{j}^{b}=\text{1}-\omega_{j}^{o}-\omega_{j}^{f}$.

#### 3.2.3. Chance-constrained stochastic programming model.

To guarantee an adequate performance, the retailer requires to ensure the service level of each channel. The service level indicates the probability that the demands in offline channel, traditional online and live streaming channels are less than or equal to the available inventory. The service level constraints are proposed as follows:


$$\;SL\left(S_{j}^{f}\right)=P\left\{S_{j}^{f}\geq {\hat{D}}_{j}^{f},\forall j\in J\right\}\geq \text{1}-\kappa^{f},$$ (11)

$$\;SL(S_{j^{\prime }j}^{o})=P\left\{\sum_{j^{\prime }\in J^{\prime }}S_{j^{\prime }j}^{o}\geq {\hat{D}}_{j}^{o},\forall j\in J\right\}\geq \text{1}-\kappa^{o},$$ (12)

$$\;SL\left(S_{j^{\prime }j}^{b}\right)=P\left\{\sum_{j^{\prime }\in J^{\prime }}S_{j^{\prime }j}^{b}\geq {\hat{D}}_{j}^{b},\forall j\in J\right\}\geq \text{1}-\kappa^{b}.$$ (13)

Constraints (11)–(13) demonstrate that the probability of shortage is no greater than a specified value $\;\kappa^{g}$,  g∈G={f,o,b}. In other words, the violation probability of unsatisfied demand cannot be over the specified value $\kappa^{g}$. A higher value of $\kappa^{g}$ will lead to a lower service level. Substitute ${\hat{D}}_{j}^{g}$ into (11)–(13), we can obtain


$$P\left\{F_{j}^{g}\left(p,p^{b},S^{g}\right)+{\left({\hat{\theta }}_{j}^{g}\right)}^{\text{T}}\xi \leq \text{0},\forall j\in J\right\}\geq \text{1}-\kappa^{g},$$ (14)

where ${\hat{\theta }}_{j}^{f}=\theta_{j}^{f}$, ${\hat{\theta }}_{j}^{o}=e\gamma^{L^{o}}\theta_{j}^{o}$, ${\hat{\theta }}_{j}^{b}=e\gamma^{L^{b}}\theta_{j}^{b}$, $\;F_{j}^{f}\left(p,p^{b},S^{f}\right)=-{\left(\alpha_{j}^{f}\right)}^{\text{T}}p+{\left(\beta_{j}\right)}^{\text{T}}p^{b}+{\left(\omega_{j}^{f}\right)}^{\text{T}}\lambda v-S^{f}$, $F_{j}^{o}\left(p,p^{b},S^{o}\right)=-{\left(\alpha_{j}^{o}\right)}^{\text{T}}p+{\left(\beta_{j}\right)}^{\text{T}}p^{b}+{\left(\omega_{j}^{o}\right)}^{\text{T}}\lambda v-S^{o}$, $F_{j}^{b}\left(p,p^{b},S^{b}\right)=-{\left(\alpha_{j}^{b}\right)}^{\text{T}}p^{b}+{\left(\beta_{j}\right)}^{\text{T}}p+{\left(\omega_{j}^{b}\right)}^{\text{T}}\lambda v-S^{b}$.

Incorporating the service level constraints (14), the chance-constrained stochastic programming model based on MQD criterion can be converted into


$$\begin{array}{c} \text{max}\;\;\;MQD_{\tau }\left(ℋ\right)\\ \text{s}.\text{t}.\;\sigma_{\text{0}}\geq Q-\sum_{j\in J}I_{j}-\sum_{g\in G\backslash \{f\}}\sum_{j\in J}S_{\text{0}j}^{g},\\ \;\;\sigma_{j}\geq I_{j}-S_{j}^{f}-\sum_{j^{\prime }\in J}S_{jj^{\prime }}^{g},\forall j\in J,\\ \;\;\varphi_{j}^{f}\geq {\hat{D}}_{j}^{f}-S_{j}^{f},\forall j\in J,\\ \;\;\varphi_{j}^{g}\geq {\hat{D}}_{j}^{g}-\sum_{j^{\prime }\in J^{\prime }}S_{j^{\prime }j}^{g},\forall j\in J,g\in G\backslash \{f\},\\ \;\;\sigma_{j}\geq \text{0},\forall j\in J,\\ \;\;\varphi_{j}^{g}\geq \text{0},\forall j\in J,g\in G,\\ \;\;P\left\{F_{j}^{g}\left(p,p^{b},S^{g}\right)+{\left({\hat{\theta }}_{j}^{g}\right)}^{\text{T}}\xi \leq \text{0},\forall j\in J\right\}\geq \text{1}-\kappa^{g},\forall g\in G,\\ \;\;p_{j}^{g},Q,I_{j},S_{j^{\prime }j}^{o},S_{j^{\prime }j}^{b},S_{j}^{f}\geq \text{0},\forall j\in J,j^{\prime }\in J^{\prime },g\in G. \end{array}$$ (15)

Assuming the probability distributions of uncertain demand could be derived from historical data, whereas it may be inaccessible to estimate the accurate probability distributions. With this motivation, we decide to construct a detailed data-driven Wasserstein based DRJCCP model considering WMQD criterion in the following section.

## 4. Data-driven distributionally robust joint chance-constrained programming

The DRJCCP version of model (15) based on imprecise probability distribution is introduced in this section. The DRJCCP model based on WMQD criterion is presented as follows:


$$\begin{array}{c} \begin{array}{c} \underset{p,p^{b},S^{𝐠},I,\sigma ,\varphi^{g}}{\text{max}}\underset{P^{\xi }\in D}{\text{inf}}\;\;{\text{MQD}}_{P^{\xi },\tau }\left(ℋ\right)\mathrm{\backslash vspace}1mm\\ \text{s}.\text{t}.\;\sigma_{\text{0}}\geq Q-\sum_{j\in J}I_{j}-\sum_{g\in G\backslash \{f\}}\sum_{j\in J}S_{\text{0}j}^{g},\mathrm{\backslash vspace}1mm\\ \;\;\sigma_{j}\geq I_{j}-S_{j}^{f}-\sum_{j^{\prime }\in J}S_{jj^{\prime }}^{g},\forall j\in J,\\ \;\;\varphi_{j}^{f}\geq {\hat{D}}_{j}^{f}-S_{j}^{f},\forall j\in J,\mathrm{\backslash vspace}1mm\\ \;\;\varphi_{j}^{g}\geq {\hat{D}}_{j}^{g}-\sum_{j^{\prime }\in J^{\prime }}S_{j^{\prime }j}^{g},\forall j\in J,g\in G\backslash \{f\},\mathrm{\backslash vspace}1mm\\ \;\;\sigma_{j}\geq \text{0},\forall j\in J,\mathrm{\backslash vspace}1mm\\ \;\;\varphi_{j}^{g}\geq \text{0},\forall j\in J,\forall g\in G,\mathrm{\backslash vspace}1mm\\ \;\;\underset{P^{{}_{\xi }}\in D}{\text{inf}}P\left\{F_{j}^{g}\left(p,p^{b},S^{g}\right)+{\left({\hat{\theta }}_{j}^{g}\right)}^{\text{T}}\xi \leq \text{0},\forall j\in J\right\}\geq \text{1}-\kappa^{g},\forall g\in G,\mathrm{\backslash vspace}1mm\\ \;\;p_{j}^{g},Q,I_{j},S_{j^{\prime }j}^{o},S_{j^{\prime }j}^{b},S_{j}^{f}\geq \text{0},\forall j\in J,j^{\prime }\in J^{\prime },g\in G. \end{array} \end{array}$$ (16)

where ${\text{inf}}_{P^{\xi }\in D}\;\;\text{M}{\text{QD}}_{P^{\xi },\tau }\left(ℋ\right)=\text{WMQ}{\text{D}}_{P^{\xi },\tau }\left(ℋ\right)$ represents the worst-case MQD, and inf(·) represents the worst-case scenario. The probability distribution ${\mathbb{P}}^{\xi }$ is assumed to be uncertain and constrained within the given uncertainty set 𝒟. The chance constraints in the formulation (16) are referred as distributional robust joint chance constraints (DRJCC). The objective function in the model (16) can be equivalently transformed into the following equation:


$$\begin{array}{c} \underset{\delta \in R^{+}}{\text{max}}\underset{P^{\xi }\in D}{\text{inf}}{\text{MQD}}_{P^{\xi },\tau }\left(ℋ\right)=\underset{\delta \in R^{+}}{\text{max}}\underset{P^{\xi }\in D}{\text{inf}}E_{P^{\xi }}\left(H\right)+\chi {\text{QD}}_{P^{\xi },\tau }\left(ℋ\right)\mathrm{\backslash vspace}1.5mm\\ =\underset{P^{\xi }\in D}{\text{inf}}\left\langle \underset{\delta \in R^{+}}{\text{max}}E_{P^{\xi }}\left(ℋ\right)-\chi \underset{\delta \in R^{+}}{\text{max}}\left\{E_{P^{\xi }}\left[(\text{1}-\tau ){\left(\delta -ℋ\right)}^{+}+\tau {\left(ℋ-\delta \right)}^{+}\right]\right\}\right\rangle \mathrm{\backslash vspace}1.5mm\\ =\underset{P^{\xi }\in D}{\text{inf}}\underset{\delta \in R^{+}}{\text{max}}\left\{E_{P^{\xi }}\left[ℋ-\chi (\text{1}-\tau ){\left(\delta -ℋ\right)}^{+}-\chi \tau {\left(ℋ-\delta \right)}^{+}\right]\right\}\mathrm{\backslash vspace}1.5mm\\ =\underset{P^{\xi }\in D}{\text{inf}}\underset{\delta \in R^{+}}{\text{max}}\left\{E_{P^{\xi }}\left[ℋ-\chi (\text{1}-\tau ){\left(\delta -ℋ\right)}^{+}-\chi \tau {\left(\delta -ℋ\right)}^{+}+\chi \tau \left(\delta -ℋ\right)\right]\right\}\mathrm{\backslash vspace}1.5mm\\ =\underset{\delta \in R^{+}}{\text{max}}\left\{\chi \tau \delta +(\text{1}-\chi \tau )\underset{P^{\xi }\in D}{\text{min}}E_{P^{\xi }}\left(ℋ\right)-\chi \underset{P^{\xi }\in D}{\text{min}}E_{P^{\xi }}\left[{\left(\delta -ℋ\right)}^{+}\right]\right\}. \end{array}$$ (17)

### 4.1. Construction of Wasserstein set

The Wasserstein metric is adopted to construct the data-driven ambiguity sets. Instead of acquiring the precise distributions of random demands, historical data are adopted to build the empirical distributions. Given a finite uncertainty demand dataset $\left\{{\hat{\xi }}_{\text{1}},{\hat{\xi }}_{\text{2}},...,{\hat{\xi }}_{\left|N\right|}\right\}$, in which ${\hat{\xi }}_{i}={\left({\hat{\xi }}_{i\text{1}},{\hat{\xi }}_{i\text{2}},...,{\hat{\xi }}_{i|J|}\right)}^{\text{T}}$ is the *i*-th historical sample of the uncertain demand, and |N| represents the number of sample data, and i∈N. An empirical probability distribution ${\hat{\mathbb{P}}}^{\xi }$ could be built so that each data point in the sample set has an equal probability of 1/1|N|\nulldelimiterspace|N|, i.e., ${\hat{\mathbb{P}}}^{\xi }=\frac{\text{1}}{\left|N\right|}\sum_{i\in N}{\bar{\delta }}_{{\hat{\xi }}_{i}}$, where ${\bar{\delta }}_{{\hat{\xi }}_{i}}$ represents the Dirac measure concentrating unit mass at ${\hat{\xi }}_{i}$. Note that ${\hat{\mathbb{P}}}^{\xi }$ serves as an estimation of the underlying true distribution ${\mathbb{P}}^{\xi }$. Intuitively, as the number of observed data points increases, ${\hat{\mathbb{P}}}^{\xi }$ gets closer to ${\mathbb{P}}^{\xi }$. The Wasserstein metric is a widely used approach to quantity this convergence.

**Definition 3.** The distance between two probability distributions ${\mathbb{P}}_{1}$ and ${\mathbb{P}}_{2}\in ℳ(\Xi )$ based on Wasserstein metric can be defined as


$$d_{W}(P_{\text{1}},P_{\text{2}})=\underset{\Pi }{\text{inf}}\{E_{\Pi }\left[\rho \left(\xi ,\hat{\xi }\right)\right]:\;\;\;\;\;\;\;\begin{array}{c} \Pi \;\text{is}\;\;\text{a}\;\;\text{joint}\;\;\text{distribution}\;\;\text{of}\;\;\xi \;\text{and}\hat{\;\;\xi }\\ \text{with}\;\;\text{marginal}\;\;\text{distributions}\;\;P_{\text{1}}\;\;\text{and}\;\;P_{\text{2}} \end{array}\;\;\;\},$$ (18)

where $E_{\Pi }\left[\rho \left(\xi ,\hat{\xi }\right)\right]=\int_{\Xi^{\text{2}}}\left\|\xi -\hat{\xi }\right\|\Pi \left(d\xi -d\hat{\xi }\right)$, ℳ(Ξ) denotes the set of all probability distributions with support set Ξ , ‖·‖ represents the norm of a vector, We adopt $l_{1}$ -norm in this paper due to its computational benefits in DRO [45].

Since we only observe a set of $\left\{{\hat{\xi }}_{\text{1}},{\hat{\xi }}_{\text{2}},...,{\hat{\xi }}_{\left|N\right|}\right\}$ of |N| i.i.d samples, the data-driven Wasserstein ambiguity sets are presented below.

**Definition 4.** The Wasserstein ambiguity set is defined as


$$𝒟=\left\{{\mathbb{P}}^{\xi }\in ℳ(\Xi )|d_{W}({\mathbb{P}}^{\xi },{\hat{\mathbb{P}}}^{\xi })\leq \mu \right\},$$ (19)

where μ denotes the Wasserstein distance between ${\mathbb{P}}^{\xi }$ and ${\hat{\mathbb{P}}}^{\xi }$. The set 𝒟 is regarded as a Wasserstein ball with a radius μ centered around the empirical distribution ${\hat{\mathbb{P}}}^{\xi }$. Hence, in a certain context, the Wasserstein ball can be interpreted as encompassing all probability distributions for which our estimation error remains within a specified threshold μ, where μ represents the maximum error we aim to protect against. A greater value of μ demonstrates that we are pursuing solutions with a higher degree of robustness. As [45] indicated, the selection of μ as a function that decreases with the sample size |N| influences the confidence that the true distribution ℙ is contained within the ambiguity set. The support set of the ξζrandom variables is given by $\;\Xi =\left\{\xi \in R^{\left|J\right|}:\underset{―}{\xi }\leq \xi \leq \overset{―}{\xi }\right\}$ with $\underset{―}{\xi }={\left({\underset{―}{\xi }}_{\text{1}},{\underset{―}{\xi }}_{\text{2}},...,{\underset{―}{\xi }}_{\left|J\right|}\right)}^{T}$ and $\overset{―}{\xi }={\left({\overset{―}{\xi }}_{\text{1}},{\overset{―}{\xi }}_{\text{2}},...,{\overset{―}{\xi }}_{\left|J\right|}\right)}^{\text{T}}$. Actually, the support set Ξ of the random variables ξ is bounded and compact. Therefore, the distribution ${\mathbb{P}}^{\xi }$ is light-tailed [45].

**Remark:** *(Relation to the SP model)* Note that when μ=0, the ambiguity set 𝒟 only includes the empirical distribution ${\hat{\mathbb{P}}}^{\xi }$, and the DRJCCP model will degenerate to the SP model with chance constraints.

The Sample Average Approximation (SAA) method is widely used in the SP approach related literature to handle uncertainty, which simply adopts the empirical distribution ${\hat{\mathbb{P}}}^{\xi }$ to approximate the true distribution ${\mathbb{P}}^{\xi }$.

Under the Wasserstein ambiguity set 𝒟, the DRJCCP (16) could be formulated as the following data-driven Wasserstein DRJCCP (WDRJCCP):


$$\begin{array}{c} \underset{p,p^{b},S^{g},I,\sigma ,\varphi^{g}}{\text{max}}\chi \tau \delta +(\text{1}-\chi \tau )\underset{P^{\xi }\in D}{\text{min}}E_{P^{\xi }}\left(ℋ\right)-\chi \underset{P^{\xi }\in D}{\text{min}}E_{P^{\xi }}\left[{\left(\delta -ℋ\right)}^{+}\right]\mathrm{\backslash vspace}1.5mm\\ \text{s}.\text{t}.\;\sigma_{\text{0}}\geq Q-\sum_{j\in J}I_{j}-\sum_{g\in G\backslash \{f\}}\sum_{j\in J}S_{\text{0}j}^{g},\mathrm{\backslash vspace}1.5mm\\ \;\;\sigma_{j}\geq I_{j}-S_{j}^{f}-\sum_{j^{\prime }\in J}S_{jj^{\prime }}^{g},\forall j\in J,\mathrm{\backslash vspace}1.5mm\\ \;\;\varphi_{j}^{f}\geq {\hat{D}}_{j}^{f}-S_{j}^{f},\forall j\in J,\mathrm{\backslash vspace}1.5mm\\ \;\;\varphi_{j}^{g}\geq {\hat{D}}_{j}^{g}-\sum_{j^{\prime }\in J^{\prime }}S_{j^{\prime }j}^{g},\forall j\in J,g\in G\backslash \{f\},\mathrm{\backslash vspace}1.5mm\\ \;\;\sigma_{j}\geq \text{0},\forall j\in J,\mathrm{\backslash vspace}1.5mm\\ \;\;\varphi_{j}^{g}\geq \text{0},\forall j\in J,g\in G,\mathrm{\backslash vspace}1.5mm\\ \;\;\underset{P^{{}_{\xi }}\in D}{\text{inf}}P\left\{F_{j}^{g}\left(p,p^{b},S^{g}\right)+{\left({\hat{\theta }}_{j}^{g}\right)}^{T}\xi \leq \text{0},\forall j\in J\right\}\geq \text{1}-\kappa^{g},\forall g\in G,\mathrm{\backslash vspace}1.5mm\\ \;\;p_{j}^{g},Q,I_{j},S_{j^{\prime }j}^{o},S_{j^{\prime }j}^{b},S_{j}^{f}\geq \text{0},\forall j\in J,j^{\prime }\in J^{\prime },g\in G. \end{array}$$ (20)

### 4.2. Model reformulation

We will derive the equivalent reformulations for the model (20) by adopting mathematical manipulations in this section. The worst-case expectation is transformed into a tractable programing problem under the ambiguity set. Therefore, the following propositions hold.

**Proposition 1.** Assuming the probability distribution ${\mathbb{P}}^{\xi }$ belongs to the ambiguity set 𝒟, and the support set $\Xi =\left\{\xi \in R^{\left|J\right|}:\underset{―}{\xi }\leq \xi \leq \overset{―}{\xi }\right\}$ is given, then the robust counterpart of ${\text{min}}_{P^{\xi }\in D}E_{P^{\xi }}\left(ℋ\right)$ could be equivalently transformed into the following model:


$$\begin{array}{c} \underset{\pi ,\vartheta_{i},\varsigma_{i},\psi_{i}}{\text{min}}\pi \mu +\frac{\text{1}}{\left|N\right|}\sum_{i\in N}\vartheta_{i}\\ \text{s}.\text{t}.\;\Delta +\sum_{g\in G}{\left(s^{g}\right)}^{\text{T}}\Theta^{g}{\hat{\xi }}_{i}+{\left(\overset{―}{\xi }-{\hat{\xi }}_{i}\right)}^{T}\varsigma_{i}-{\left(\underset{―}{\xi }-{\hat{\xi }}_{i}\right)}^{T}\psi_{i}\leq \vartheta_{i},\forall i\in N,\\ \;\;\;\left\|\sum_{g\in G}\Theta^{g}s^{g}-\varsigma_{i}+\psi_{i}\right\|\leq \pi ,\forall i\in N,\\ \;\;\;\varsigma_{i}\geq \text{0},\psi_{i}\geq \text{0},\forall i\in N, \end{array}$$ (21)

where ‖·‖ represents the dual norm, and π, $\varsigma_{i}$, $\psi_{i}$ are the dual variables/vectors, $\vartheta_{i}$ is an auxiliary variable, $Y^{f}=-{𝐀}^{f}p+𝐁p^{b}+\Omega^{f}\lambda v-S^{f}$, $Y^{o}=-{𝐀}^{o}p+𝐁p^{b}+\Omega^{o}\lambda v-S^{o}$, $Y^{b}=-{𝐀}^{b}p^{b}+𝐁p+\Omega^{b}\lambda v-S^{b}$, $V=-h_{0}\sigma_{0}-wQ-nv^{2}$, .

**Proof.** Please see S1 Appendix.

**Proposition 2.** The probability distribution ${\mathbb{P}}^{\xi }$ is considered to be within the ambiguity set 𝒟, and the support set of stochastic variables $\;\Xi =\left\{\xi \in R^{\left|J\right|}:\underset{―}{\xi }\leq \xi \leq \overset{―}{\xi }\right\}$ has been defined, then the worst-case expectation $(\text{1}-\chi \tau ){\text{min}}_{P^{\xi }\in D}E_{P^{\xi }}\left(ℋ\right)-\chi {\text{min}}_{P^{\xi }\in D}E_{P^{\xi }}\left[{\left(\delta -ℋ\right)}^{+}\right]$ in model (20) is equal to the following formulations:


$$\;\begin{array}{c} \underset{\pi^{\prime },{\vartheta^{\prime }}_{i},{\varsigma^{\prime }}_{i},{\psi^{\prime }}_{i}}{\text{min}}\;\;\;\pi^{\prime }\mu +\frac{\text{1}}{\left|N\right|}\sum_{i\in N}{\vartheta^{\prime }}_{i}\mathrm{\backslash vspace}1mm\\ \text{s}.\text{t}.\;{\Delta^{{}^{\prime }}}_{r}-{{𝒬}_{r}}^{\text{T}}{\hat{\xi }}_{i}+{\left(\overset{―}{\xi }-{\hat{\xi }}_{i}\right)}^{\text{T}}{\varsigma^{\prime }}_{i}-{\left(\underset{―}{\xi }-{\hat{\xi }}_{i}\right)}^{\text{T}}{\psi^{{}^{\prime }}}_{i}\leq {\vartheta^{\prime }}_{i},\forall i\in N,r\in \{\text{1},\text{2}\},\mathrm{\backslash vspace}1.5mm\\ \;\;\;\left\|Q_{r}-{\varsigma^{\prime }}_{i}+{\psi^{\prime }}_{i}\right\|\leq \pi^{\prime },\forall i\in N,r\in \{\text{1},\text{2}\},\mathrm{\backslash vspace}1.5mm\\ \;\;\;{\varsigma^{\prime }}_{i}\geq \text{0},\psi_{i}\geq \text{0},\forall i\in N. \end{array}$$ (22)

where $\pi^{\prime }$, $\varsigma_{i}^{\prime }$, $\psi_{i}^{\prime }$ are the dual variables/vectors, $\vartheta_{i}^{\prime }$ is an auxiliary variable. ${\Delta^{\prime }}_{\text{1}}=(\text{1}-\chi \tau +\chi )\Delta +\chi \delta$, ${\Delta^{\prime }}_{\text{2}}=(\text{1}-\chi \tau )\Delta$, ${𝒬}_{\text{1}}=(\text{1}-\chi \tau +\chi sum_{g\in G}\Theta^{g}s^{g}$, ${𝒬}_{\text{2}}=(\text{1}-\chi \tau sum_{g\in G}\Theta^{g}s^{g}$.

**Proof.** Please see S1 Appendix.

In addition, to cope with the uncertain distributions in the DRJCCs, a conservative approximation of chance constraints is obtained by adopting the worst-case CVaR constraints [73]. And then, by using the duality theory, the approximation set can be derived. The commonly adopted approximation approaches for the DRJCC, such as Bonferroni inequality and the approach proposed by [74], are built on inequalities from probability theory, which are unnecessarily tight. However, the worst-case CVaR constraint has exact tractable reformulations in terms of Linear Matrix Inequalities (LMIs), which provide a tight convex approximation for DRJCC [73]. With $F^{g}\left(p,p^{b},S^{g}\right)={\text{max}}_{j\in J}\left\{F_{j}^{g}\left(p,p^{b},S^{g}\right)+{\left({\hat{\theta }}_{j}^{g}\right)}^{\text{T}}\xi \right\}$. The DRJCCs in model (20) can be transformed into the following constraints:


$$\underset{P^{{}_{\xi }}\in D}{\text{inf}}P\left\{F^{g}\left(p,p^{b},S^{g}\right)\leq \text{0}\right\}\geq \text{1}-\kappa^{g},\forall g\in G.$$ (23)

By using a constraint that includes the CVaR at level $\kappa^{g}$ with respect to ${\mathbb{P}}^{\xi }$, Constraints (23) can be conservatively approximated, which can be expressed mathematically by the following implication:


$$\begin{array}{c} \underset{P^{\xi }\in D}{\text{sup}}\text{CVa}{\text{R}}_{\tau^{g}}\left(F^{g}\left(p,p^{b},S^{g}\right)\right)\leq \text{0}\\ \Rightarrow \underset{P^{\xi }\in D}{\text{min}}P^{\xi }\left(F^{g}\left(p,p^{b},S^{g}\right)\leq \text{0}\right)\geq \text{1}-\kappa^{g},\forall g\in G, \end{array}$$ (24)

Constraints (24) shows that the CVaR formulation is adequate to impose the DRJCC. It is a conservative approximation, as the CVaR takes the violation magnitude into account, thereby enforcing the constraints with a probability that exceeds a priori specified level. By adopting the definition of CVaR, the left-hand side of (24) can be expressed as:


$$\underset{P^{\xi }\in D}{\text{max}}\underset{\iota^{g}\in R^{+}}{\text{min}}\;\iota^{g}+\frac{\text{1}}{\kappa^{g}}E_{P^{\xi }}\left[{\left(F^{g}\left(p,p^{b},S^{g}\right)-\iota^{g}\right)}^{+}\right],\forall g\in G,$$ (25)

where $\iota^{g}\in R^{+}$ is an auxiliary variable. After changing the order of the optimization operators, Eq. (25) can be transformed into:


$$\underset{\iota^{g}\in R^{+}}{\text{min}}\;\iota^{g}+\frac{\text{1}}{\kappa^{g}}\underset{P^{\xi }\in D}{\text{max}}E_{P^{\xi }}\left[{\left(F^{g}\left(p,p^{b},S^{g}\right)-\iota^{g}\right)}^{+}\right],\forall g\in G.$$ (26)

Following the mathematical procedure, we equivalently drop the min operators and add auxiliary variables, and then, the CVaR approximation of the DRJCC is obtained.

**Proposition 3.** With the given support set $\;\Xi =\left\{\xi \in R^{\left|J\right|}:\underset{―}{\xi }\leq \xi \leq \overset{―}{\xi }\right\}$, the DRJCC based on Wasserstein set is equivalent to the following set:


$$\left\{ \;\;\begin{array}{c} \iota^{g}+\frac{\text{1}}{\kappa^{g}}\left(\zeta^{g}\mu +\frac{\text{1}}{\left|N\right|}\sum_{i\in N}t_{i}^{g}\right)\leq \text{0},\forall g\in G,\mathrm{\backslash vspace}1mm\\ t_{i}^{g}\geq {\left[F_{j}^{g}\left(p,p^{b},S^{𝐠}\right)-\iota^{g}+{\left({\overset{⌢}{\theta }}_{j}^{g}\right)}^{\text{T}}{\hat{\xi }}_{i}+{\left(\overset{―}{\xi }-{\hat{\xi }}_{i}\right)}^{T}k_{ij}^{g}-{\left(\underset{―}{\xi }-{\hat{\xi }}_{i}\right)}^{\text{T}}u_{ij}^{g}\right]}^{+},\mathrm{\backslash vspace}1mm\\ \forall i\in N,j\in J,g\in G,\mathrm{\backslash vspace}1mm\\ \left\|{\overset{⌢}{\theta }}_{j}^{g}-k_{ij}^{g}+u_{ij}^{g}\right\|\leq \zeta^{g},\forall i\in N,g\in G,\mathrm{\backslash vspace}1mm\\ \zeta^{g}\geq \text{0},\;k_{ij}^{g}\geq \text{0},u_{ij}^{g}\geq \text{0},\forall i\in N,j\in J,g\in G. \end{array}\right.\;$$ (27)

**Proof.** Please see S1 Appendix.

With Proposition 2 and 3, model (20) can be reformulated into the following equivalent formulation:


$$\;\begin{array}{c} \text{max}\;\chi \tau \delta -\pi^{\prime }\mu -\frac{\text{1}}{\left|N\right|}\sum_{i\in N}{\vartheta^{\prime }}_{i}\\ \text{s}.\text{t}.\;\;\;\Delta_{r}^{{}^{\prime }}+{𝒬}_{r}^{\text{T}}\;{\hat{\xi }}_{i}+{\left(\overset{―}{\xi }-{\hat{\xi }}_{i}\right)}^{\text{T}}{\varsigma^{{}^{\prime }}}_{i}-{\left(\underset{―}{\xi }-{\hat{\xi }}_{i}\right)}^{\text{T}}\psi_{i}\leq {\vartheta^{\prime }}_{i},\forall i\in N,\forall r\in \{\text{1},\text{2}\},\mathrm{\backslash vspace}1.5mm\\ \;\;\;\left\|{𝒬}_{r}-{\varsigma^{{}^{\prime }}}_{i}+{\psi^{\prime }}_{i}\right\|\leq \pi^{\prime },\forall i\in N,r\in \{\text{1},\text{2}\},\mathrm{\backslash vspace}1.5mm\\ \;\;\;\iota^{g}+\frac{\text{1}}{\kappa^{g}}\left(\zeta^{g}\mu +\frac{\text{1}}{\left|N\right|}\sum_{i\in N}t_{i}^{g}\right)\leq \text{0},\;\forall g\in G,\\ \;\;\;t_{i}^{g}\geq {\left[F_{j}^{g}\left(p,p^{b},S^{g}\right)-\iota^{g}+{\left(\theta_{j}^{g}\right)}^{\text{T}}{\hat{\xi }}_{i}+{\left(\overset{―}{\xi }-{\hat{\xi }}_{i}\right)}^{\text{T}}k_{ij}^{g}-{\left(\underset{―}{\xi }-{\hat{\xi }}_{i}\right)}^{\text{T}}u_{ij}^{g}\right]}^{+},\mathrm{\backslash vspace}1.5mm\\ \;\;\;\forall i\in N,j\in J,g\in G\mathrm{\backslash vspace}1.5mm\\ \;\;\;\left\|{\hat{\theta }}_{j}^{g}-k_{ij}^{g}+u_{ij}^{g}\right\|\leq \zeta^{g},\forall i\in N,j\in J,g\in G,\\ \;\;\;\sigma_{\text{0}}\geq Q-1^{\text{T}}I-\sum_{g\in G\backslash \{f\}}1^{\text{T}}S_{\text{0}}^{g},\mathrm{\backslash vspace}1.5mm\\ \;\;\;\sigma_{j}\geq I_{j}-S_{j}^{f}-\sum_{j^{\prime }\in J}S_{jj^{\prime }}^{g},\forall j\in J,\mathrm{\backslash vspace}1.5mm\\ \;\;\;p_{j}^{g},Q,I_{j},S_{j^{\prime }j}^{o},S_{j^{\prime }j}^{b},S_{j}^{f},\sigma_{\text{0}},\sigma_{j},{\varsigma^{\prime }}_{i},{\psi^{\prime }}_{i},\zeta^{g},k_{ij}^{g},u_{ij}^{g}\geq \text{0},\mathrm{\backslash vspace}1.5mm\\ \;\;\;\forall i\in N,j\in J,j^{\prime }\in J^{\prime },g\in G. \end{array}$$ (28)

Note that model (28) is a non-linear program due to the bilinear terms ${\left(p^{g}\right)}^{\text{T}}S^{g}$ in the expression of $\Delta_{r}^{{}^{\prime }}$. Therefore, according to [58], the piecewise affine relaxation is adopted to approximate model (28).

### 4.3. Linearization of bilinear terms

We assume the continuous variables $p_{j}^{g}$, $S_{j}^{f}$ $S_{j^{\prime }j}^{o}$, $S_{j^{\prime }j}^{b}$ to be bounded, i.e., $\;p_{j}^{g}\in [{\underset{―}{p}}_{j}^{g},{\overset{―}{p}}_{j}^{g}]$, $\;S_{j}^{f}\in [{\underset{―}{S}}_{j}^{f},{\overset{―}{S}}_{j}^{f}]$, $\;S_{j^{\prime }j}^{o}\in [{\underset{―}{S}}_{j^{\prime }j}^{o},{\overset{―}{S}}_{j^{\prime }j}^{o}]$, $\;S_{j^{\prime }j}^{b}\in [{\underset{―}{S}}_{j^{\prime }j}^{b},{\overset{―}{S}}_{j^{\prime }j}^{b}]$. Define $M_{j}^{g}$ ($M_{j}^{g}\geq \text{1}$) to be partitioning factor for $p_{j}^{g}$. The region of $p_{j}^{g}$ is partitioned into $Z_{j}^{g}$ intervals with the increment of $\;M_{j}^{g}=\left({\overset{―}{p}}_{j}^{g}-{\underset{―}{p}}_{j}^{g}\right)/Z_{j}^{g}$. According to the classic McCormick relaxation [75], the maximization difference between bilinear terms and the corresponding $M_{j}^{g}$ envelopes can be expressed as $\;R_{j}^{f}=M_{j}^{f}\left({\overset{―}{S}}_{j}^{f}-{\underset{―}{S}}_{j}^{f}\right)/\text{4}$, $\;R_{j^{\prime }j}^{o}=M_{j}^{o}\left({\overset{―}{S}}_{j^{\prime }j}^{o}-{\underset{―}{S}}_{j^{\prime }j}^{o}\right)/\text{4}$, $\;R_{j^{\prime }j}^{b}=M_{j}^{b}\left({\overset{―}{S}}_{j^{\prime }j}^{b}-{\underset{―}{S}}_{j^{\prime }j}^{b}\right)/\text{4}$, respectively. Auxiliary binary variables $y_{jl}^{f}$, $l=\text{1},\text{2},...,Z_{j}^{f}$, $y_{j^{\prime }jl}^{o}$, $l=\text{1},\text{2},...,Z_{j}^{o}$, $y_{j^{\prime }jl}^{b}$, $l=\text{1},\text{2},...,Z_{j}^{b}$ are introduced, and ${\hat{S}}_{jl}^{f}$, $l=\text{1},\text{2},...,Z_{j}^{f}$, ${\hat{S}}_{j^{\prime }jl}^{o}$, $l=\text{1},\text{2},...,Z_{j}^{o}$, ${\hat{S}}_{j^{\prime }jl}^{b}$, $l=\text{1},\text{2},...,Z_{j}^{b}$ are introduced as continuous switches. The constraints $\;\Delta_{r}^{{}^{\prime }}+{𝐐}_{r}^{\text{T}}{\hat{\xi }}_{i}+{\left(\overset{―}{\xi }-{\hat{\xi }}_{i}\right)}^{T}{\varsigma^{\prime }}_{i}-{\left(\underset{―}{\xi }-{\hat{\xi }}_{i}\right)}^{T}\psi_{i}^{\prime }\leq {\vartheta^{\prime }}_{i},\forall i\in N,\forall r\in \{\text{1},\text{2}\}$, in model (28) can be converted into:


$$\left\{ \begin{array}{c} \left(1-\chi \tau +\chi \right)\left(c^{T}I+\sum_{j\in J}{\left(m_{j}^{o}\right)}^{T}S_{j}^{o}+\sum_{j\in J}{\left(m_{j}^{b}\right)}^{T}S_{j}^{b}+\sum_{g\in G}{\left(s^{g}\right)}^{T}Y^{g}+h^{T}\sigma -V+\sum_{g\in G}{\left(s^{g}\right)}^{T}\Theta^{g}{\hat{\xi }}_{i}\right)+\chi \delta \\ +{\left(\overset{―}{\xi }-{\hat{\xi }}_{i}\right)}^{T}{\varsigma^{\prime }}_{i}-{\left(\underset{―}{\xi }-{\hat{\xi }}_{i}\right)}^{T}{\psi_{i}}^{\prime }+\left(1-\chi \tau +\chi \right)\left(\sum_{g\in G\backslash \left\{b\right\}}1^{T}z^{g}+\left(1-\epsilon \right)1^{T}z^{b}\right)\leq {\vartheta^{\prime }}_{i},\forall i\in N,\\ \left(1-\chi \tau \right)\left(c^{T}I+\sum_{j\in J}{\left(m_{j}^{o}\right)}^{T}S_{j}^{o}+\sum_{j\in J}{\left(m_{j}^{b}\right)}^{T}S_{j}^{b}+\sum_{g\in G}{\left(s^{g}\right)}^{T}Y^{g}+h^{T}\sigma -V+\sum_{g\in G}{\left(s^{g}\right)}^{T}\Theta^{g}{\hat{\xi }}_{i}\right)\\ +{\left(\overset{―}{\xi }-{\hat{\xi }}_{i}\right)}^{T}{\varsigma^{\prime }}_{i}-{\left(\underset{―}{\xi }-{\hat{\xi }}_{i}\right)}^{T}{\psi_{i}}^{\prime }+\left(1-\chi \tau \right)\left(\sum_{g\in G\backslash \;\left\{b\right\}}1^{T}z^{g}+\left(1-\epsilon \right)1^{T}z^{b}\right)\leq {\vartheta^{\prime }}_{i},\forall i\in N,\\ {\underset{―}{p}}_{j}^{f}+M_{j}^{f}{\left({\overset{⌢}{Z}}_{j}^{f}\right)}^{T}y_{j}^{f}\leq p_{j}^{f}\leq {\underset{―}{p}}_{j}^{f}+M_{j}^{f}{\left(Z_{j}^{f}\right)}^{T}y_{j}^{f},\forall j\in J,\\ {\underset{―}{p}}_{j}^{o}+M_{j}^{o}\sum_{j^{\prime }\in J^{\prime }}{\left({\overset{⌢}{Z}}_{j}^{o}\right)}^{T}y_{j^{\prime }j}^{o}\leq p_{j}^{o}\leq {\underset{―}{p}}_{j}^{o}+M_{j}^{o}\sum_{j^{\prime }\in J^{\prime }}{\left(Z_{j}^{o}\right)}^{T}y_{j^{\prime }j}^{o},\forall j\in J,\\ {\underset{―}{p}}_{j}^{b}+M_{j}^{b}\sum_{j^{\prime }\in J^{\prime }}{\left({\overset{⌢}{Z}}_{j}^{b}\right)}^{T}y_{j^{\prime }j}^{b}\leq p_{j}^{b}\leq {\underset{―}{p}}_{j}^{b}+M_{j}^{b}\sum_{j^{\prime }\in J^{\prime }}{\left(Z_{j}^{b}\right)}^{T}y_{j^{\prime }j}^{b},\forall j\in J,\\ 1^{T}y_{j}^{f}=1,\forall j\in J,\\ 1^{T}y_{j^{\prime }j}^{g}=1,\forall j\in J,j^{\prime }\in J^{\prime },g\in G\backslash \;\left\{f\right\},\\ y_{jl}^{f},y_{j^{\prime }jl}^{o},y_{j^{\prime }jl}^{b}\in \left\{0,1\right\},l=1,2,...,Z_{j}^{g},\forall j\in J,j^{\prime }\in J^{\prime },\\ S_{j}^{f}={\underset{―}{S}}_{j}^{f}+1^{T}{\overset{⌢}{S}}_{j}^{f},\forall j\in J,\\ S_{j^{\prime }j}^{g}={\underset{―}{S}}_{j^{\prime }j}^{g}+1^{T}{\overset{⌢}{S}}_{j^{\prime }j}^{g},\forall j\in J,j^{\prime }\in J^{\prime },g\in G\backslash \;\left\{f\right\},\\ 0\leq {\overset{⌢}{S}}_{jl}^{f}\leq \left({\overset{―}{S}}_{j}^{f}-{\underset{―}{S}}_{j}^{f}\right)y_{jl}^{f},l=1,2,...,Z_{j}^{f},\forall j\in J,\\ 0\leq {\overset{⌢}{S}}_{j^{\prime }jl}^{g}\leq \left({\overset{―}{S}}_{j^{\prime }j}^{g}-{\underset{―}{S}}_{j^{\prime }j}^{g}\right)y_{j^{\prime }jl}^{g},l=1,2,...,Z_{j}^{g},\forall j\in J,j^{\prime }\in J^{\prime },g\in G\backslash \;\left\{f\right\}. \end{array}\right.$$ (29)

The elements in vectors $\;z^{g}$ is given as

$\;z_{j}^{f}=\text{max}\left\{\;\;\;\;\begin{array}{c} -p_{j}^{f}{\underset{―}{S}}_{j}^{f}-\left({\underset{―}{p}}_{j}^{f}1^{T}{\hat{S}}_{j}^{f}+M_{j}^{f}{\left(Z_{j}^{f}\right)}^{\text{T}}{\hat{S}}_{j}^{f}\right)+R_{j}^{f},-p_{j}{\overset{―}{S}}_{j}^{f}\\ -\left({\underset{―}{p}}_{j}^{f}1^{\text{T}}{\hat{S}}_{j}^{f}-{\underset{―}{p}}_{j}^{f}\left({\overset{―}{S}}_{j}^{f}-{\underset{}{S}}_{j}^{f}\right)1^{\text{T}}y_{j}^{f}+M_{j}^{f}{\left({\hat{Z}}_{j}^{f}\right)}^{T}{\hat{S}}_{j}^{f}-M_{j}^{f}\left({\overset{―}{S}}_{j}^{f}-{\underset{―}{S}}_{j}^{f}\right){\left({\hat{Z}}_{j}^{f}\right)}^{\text{T}}y_{j}^{f}\right)+R_{j}^{f} \end{array}\;\;\;\right\}$, $\;z_{j}^{o}=\text{max}\left\{\;\;\;\;\begin{array}{c} -p_{j}^{o}\sum_{j^{\prime }\in J^{\prime }}{\underset{―}{S}}_{j^{\prime }j}^{o}-\left({\underset{―}{p}}_{j}^{o}1^{\text{T}}{\hat{S}}_{j}^{o}+M_{j}^{o}{\left(Z_{j}^{o}\right)}^{\text{T}}{\hat{S}}_{j}^{o}\right)+1^{\text{T}}R_{j}^{o},-p_{j}^{o}\sum_{j^{\prime }\in J^{\prime }}{\overset{―}{S}}_{j^{\prime }j}^{o}\\ -\left({\underset{―}{p}}_{j}^{o}1^{\text{T}}{\hat{S}}_{j}^{o}-{\underset{―}{p}}_{j}^{o}\sum_{j^{\prime }\in J^{\prime }}\left({\overset{―}{S}}_{j^{\prime }j}^{o}-{\underset{―}{S}}_{j^{\prime }j}^{o}\right)1^{T}y_{j^{\prime }j}^{o}+M_{j}^{o}{\left({\hat{Z}}_{j}^{o}\right)}^{\text{T}}{\hat{S}}_{j}^{o}-M_{j}^{o}\sum_{j^{\prime }\in J^{\prime }}\left({\overset{―}{S}}_{j^{\prime }j}^{o}-{\underset{―}{S}}_{j^{\prime }j}^{o}\right){\left({\hat{Z}}_{j}^{o}\right)}^{\text{T}}y_{j^{\prime }j}^{o}\right)+1^{\text{T}}R_{j}^{o} \end{array}\right\}$, $\;z_{j}^{b}=\text{max}\left\{\;\;\;\begin{array}{c} -p_{j}^{b}\sum_{j^{\prime }\in J^{\prime }}{\underset{―}{S}}_{j^{\prime }j}^{b}-\left({\underset{―}{p}}_{j}^{b}1^{\text{T}}{\hat{S}}_{j}^{b}+M_{j}^{b}{\left(Z_{j}^{b}\right)}^{\text{T}}{\hat{S}}_{j}^{b}\right)+1^{\text{T}}R_{j}^{b},-p_{j}^{b}\sum_{j^{\prime }\in J^{\prime }}{\overset{―}{S}}_{j^{\prime }j}^{b}\\ -\left({\underset{―}{p}}_{j}^{b}1^{\text{T}}{\hat{S}}_{j}^{b}-{\underset{―}{p}}_{j}^{b}\sum_{j^{\prime }\in J^{\prime }}\left({\overset{―}{S}}_{j^{\prime }j}^{b}-{\underset{―}{S}}_{j^{\prime }j}^{b}\right)1^{\text{T}}y_{j^{\prime }j}^{b}+M_{j}^{b}{\left({\hat{Z}}_{j}^{b}\right)}^{\text{T}}{\hat{S}}_{j}^{b}-M_{j}^{b}\sum_{j^{\prime }\in J^{\prime }}\left({\overset{―}{S}}_{j^{\prime }j}^{b}-{\underset{―}{S}}_{j^{\prime }j}^{b}\right){\left({\hat{Z}}_{j}^{b}\right)}^{\text{T}}y_{j^{\prime }j}^{b}\right)+1^{\text{T}}R_{j}^{b} \end{array}\right\}$, where $\;Z_{j}^{g}={\left(\text{1},\text{2},...,Z_{j}^{g}\right)}^{\text{T}}$, $\;{\hat{Z}}_{j}^{g}={\left(\text{0},\text{1},...,Z_{j}^{g}-\text{1}\right)}^{\text{T}}$, $\;y_{j}^{f}={\left(y_{j\text{1}}^{f},y_{j\text{2}}^{f},...,y_{jZ_{j}^{f}}^{f}\right)}^{\text{T}}$ $\;y_{j^{\prime }j}^{o}=(y_{j^{\prime }j\text{1}}^{o},y_{j^{\prime }j\text{2}}^{o}\;,...,{\;y_{j^{\prime }jZ_{j}^{o}}^{o})}^{\text{T}}$, $\;y_{j^{\prime }j}^{b}={\left(y_{j^{\prime }j\text{1}}^{b},y_{j^{\prime }j\text{2}}^{b},...,y_{j^{\prime }jZ_{j}^{b}}^{b}\right)}^{\text{T}}$, $\;{\hat{S}}_{j}^{f}={\left({\hat{S}}_{j\text{1}}^{f},{\hat{S}}_{j\text{2}}^{f},...,{\hat{S}}_{jZ_{j}^{f}}^{f}\right)}^{\text{T}}$, $\;{\hat{S}}_{j}^{o}={\left(\sum_{j^{\prime }\in \left|J^{\prime }\right|}{\hat{S}}_{j^{\prime }j\text{1}}^{o},\sum_{j^{\prime }\in \left|J^{\prime }\right|}{\hat{S}}_{j^{\prime }j\text{2}}^{o},...,\sum_{j^{\prime }\in \left|J^{\prime }\right|}{\hat{S}}_{j^{\prime }jZ_{j}^{o}}^{o}\right)}^{\text{T}}$, $\;{\hat{S}}_{j^{\prime }j}^{o}={\left({\hat{S}}_{j^{\prime }j\text{1}}^{o},{\hat{S}}_{j^{\prime }j\text{2}}^{o},...,{\hat{S}}_{j^{\prime }jZ_{j}^{o}}^{o}\right)}^{\text{T}}$, $\;{\overset{⌢}{S}}_{j}^{b}={\left(\sum_{j^{\prime }\in \left|J^{\prime }\right|}{\hat{S}}_{j^{\prime }j\text{1}}^{b},\sum_{j^{\prime }\in \left|J^{\prime }\right|}{\hat{S}}_{j^{\prime }j\text{2}}^{b},...,\sum_{j^{\prime }\in \left|J^{\prime }\right|}{\hat{S}}_{j^{\prime }jZ_{j}^{b}}^{b}\right)}^{\text{T}}$, $\;{\hat{S}}_{j^{\prime }j}^{b}={\left({\hat{S}}_{j^{\prime }j\text{1}}^{b},{\hat{S}}_{j^{\prime }j\text{2}}^{b},...,{\hat{S}}_{j^{\prime }jZ_{j}^{b}}^{b}\right)}^{\text{T}}$, $\;R_{j}^{o}=\left(R_{\text{0}j}^{o},R_{\text{1}j}^{o},...,R_{\left|J^{\prime }\right|j}^{o}\right)$, $\;R_{j}^{b}=\left(R_{\text{0}j}^{b},R_{\text{1}j}^{b},...,R_{\left|J^{\prime }\right|j}^{b}\right)$.

Therefore, model (28) can be formulated as follows:


$$\begin{array}{cc} & \text{max}\;\chi \tau \delta -\pi^{\prime }\mu -\frac{\text{1}}{\left|N\right|}\sum_{i\in N}{\vartheta^{\prime }}_{i}\\ & \text{s}.\text{t}.\;\;\;\left\|{𝒬}_{r}-{\varsigma^{\prime }}_{i}+{\psi^{\prime }}_{i}\right\|\leq \pi^{\prime },\forall i\in N,r\in \{\text{1},\text{2}\},\\ & \;\;\;\;\;\;\;\iota^{g}+\frac{\text{1}}{\kappa^{g}}\left(\zeta^{g}\mu +\frac{\text{1}}{\left|N\right|}\sum_{i\in N}t_{i}^{g}\right)\leq \text{0},\;\forall g\in G,\\ & \;\;\;\;\;\;\;t_{i}^{g}\geq {\left[F_{j}^{g}\left(p,p^{b},S^{g}\right)-\iota^{g}+{\left({\overset{⌢}{\theta }}_{j}^{g}\right)}^{\text{T}}{\hat{\xi }}_{i}+{\left(\overset{―}{\xi }-{\hat{\xi }}_{i}\right)}^{\text{T}}k_{ij}^{g}-{\left(\underset{―}{\xi }-{\hat{\xi }}_{i}\right)}^{\text{T}}u_{ij}^{g}\right]}^{+},\\ & \;\;\;\;\;\;\;\forall i\in N,j\in J,g\in G,\\ & \;\;\;\;\;\;\;\left\|{\hat{\theta }}_{j}^{g}-k_{ij}^{g}+u_{ij}^{g}\right\|\leq \zeta^{g},\forall i\in N,j\in J,g\in G,\\ & \;\;\;\;\;\;\;\sigma_{\text{0}}\geq Q-1^{\text{T}}I-\sum_{g\in G\backslash \{f\}}1^{\text{T}}S_{\text{0}}^{g},\\ & \;\;\;\;\;\;\;\;\sigma_{j}\geq I_{j}-S_{j}^{f}-\sum_{j^{\prime }\in J}S_{jj^{\prime }}^{g},\forall j\in J,\\ & \;\;\;\;\;\;\;(29)\\ & \;\;\;\;\;\;\;\;p_{j}^{g},Q,I_{j},S_{j^{\prime }j}^{o},S_{j^{\prime }j}^{b},S_{j}^{f},\sigma_{\text{0}},\sigma_{j},{\varsigma^{\prime }}_{i},{\psi^{\prime }}_{i},\zeta^{g},k_{ij}^{g},u_{ij}^{g}\geq \text{0},\\ & \;\;\;\;\;\;\;\forall i\in N,j\in J,j^{\prime }\in J^{\prime },g\in G.\;\;\;\;\; \end{array}$$ (30)

### 4.4. Methodology step

In terms of the methodological steps, a chance-constrained stochastic programming model based on MQD criterion is first introduced. Furthermore, the Wasserstein metric is adopted to establish the data-driven ambiguity set, and a data-driven WDRJCCP model under inaccurate probability distribution is thus established. With the designed ambiguity set, the WDRJCCP is transferred into a bilinear optimization problem, and approximated into a tractable formulation by adopting piecewise affine relaxation, which can be solved by state-of-art solvers, such as CPLEX and Gurobi.

## 5. Numerical studies

We present the numerical experiments to verify the practical applicability of the developed model and examine the effectiveness of the solution methodology. The numerical study is conducted using the related data provided by an omnichannel retailer that sells seasonal products. However, due to business confidentiality, the actually market data are not available. Therefore, reasonable parameter values are assigned to the associated parameters with appropriate modifications referring to the dataset in [26]. The numerical experiments are implemented in ILOG’s CPLEX 12.9 solver on a desktop with Intel Core i7 3.60 GHz processor and 16.0 GB RAM.

### 5.1. Instance generation

The retailer operates stores in each of the 5 regions, i.e., |J|=5. We suppose the mean of each region’s aggregate demand is 10000, where the percentage of traditional online and live streaming demand are denoted by $\;\theta_{j}^{o}\in [\text{0},\text{0}.\text{4}]$ and $\;\theta_{j}^{b}\in [\text{0},\text{0}.\text{3}]$, respectively. Self-price elasticity $\;\alpha_{j}^{g}\in [\text{0},\text{1}]$ and cross-price elasticity $\beta_{j}\in [0,1]$. Delivery times in channel ***o*** and ***b*** are $\;L^{o}\in [\text{1},\text{2}]$ and $\;L^{b}\in [\text{2},\text{3}]$. Table 3 gives the related parameters, with each parameter is randomly generated from its respective range. Note that these settings satisfy certain specified conditions: (i) transporting a single parcel for traditional online order or live streaming order incurs higher costs compared to bulk shipping during inventory replenishment. In addition, the fulfillment costs for traditional online orders are more expensive than that for live streaming orders, since live streaming order arrivals tend to be more concentrated, thus, $\;m_{j^{\prime }j}^{o}>m_{j^{\prime }j}^{b}>c_{j}$; (ii) $\;s_{j}^{f}>s_{j}^{o}>s_{j}^{b}$, which signifies that the inventory in store j is firstly used to fulfill in-store demand, and then traditional online demand; (iii) $h_{j}>h_{0}$, where j∈J, which indicates that the inventory holding cost in the DC is lower than that in stores, as the usage cost of DC is lower than that of stores; (iv) $\;L_{j}^{b}>L_{j}^{o}$, since live streaming customers have lower inherent expectations for delivery time compared to traditional e-commerce customers, and the quantity of orders in live streaming channel is always unpredictable.

**Table 3:** Relative parameter distributions.

Parameter	value	Parameter	Value
w	[80,120]	$c_{j}$	[2,4]
$\;m_{j^{\prime }j}^{o}$	${\underset{―}{m}}_{j^{\prime }j}^{o}+a/L^{o}$	${\underset{―}{m}}_{j^{\prime }j}^{o}$	[6,7]
$\;m_{j^{\prime }j}^{b}$	${\underset{―}{m}}_{j^{\prime }j}^{b}+a/L^{b}$	${\underset{―}{m}}_{j^{\prime }j}^{o},j^{\prime }\neq j$	${\underset{―}{m}}_{jj}^{o}+\epsilon ,\epsilon \in [\text{1},\text{2}]$
$\;{\underset{―}{m}}_{\text{0}j}^{o}$	[8,9]	$\;{\underset{―}{m}}_{jj}^{b}$	[4,5]
${\underset{―}{m}}_{\text{0}j}^{b}$	[7,8]	${\underset{―}{m}}_{j^{\prime }j}^{b},j^{\prime }\neq j$	${\underset{―}{m}}_{jj}^{b}+\epsilon ,\epsilon \in [\text{1},\text{2}]$
a	[2,4]	n	[30000,80000]
$s_{j}^{f}$	[80,100]	$\;s_{j}^{o}$	[60,80]
$s_{j}^{b}$	[40,60]	$h_{j}$	[50,70]
$\lambda_{j}$	[0,1]	ε	[0.05,0.5]
$\;\omega_{j}^{o}$	(0,0.3]	$\;\omega_{j}^{f}$	(0,0.3]
$\;\kappa^{g}$	0.1		

### 5.2. Computational results

When τ=0.2, χ=0.5, the relative computational results are displayed in Table 4. It can be seen that the optimal decisions could be solved within short CPU times. The average prices in offline, traditional online, and live streaming channels are 292.1, 292.1 and 290.4, respectively. The computational results show that the prices in offline and traditional online channels are slightly higher than that in live streaming channel. In addition, the ordering quantity is 8316, and the replenishment quantity is 1157. Note that Obj represents the objective value and Exp represents the expected profit, which are also used in the subsequent analyses.

**Table 4:** The optimal solutions.

Obj	Exp	Ordering quantity	Replenishment quantity	Price	CPU times(s)
957,886.56	924,650.5	8316	1157	[292.1,292.1,290.4]	8.54

**Fig 2:**
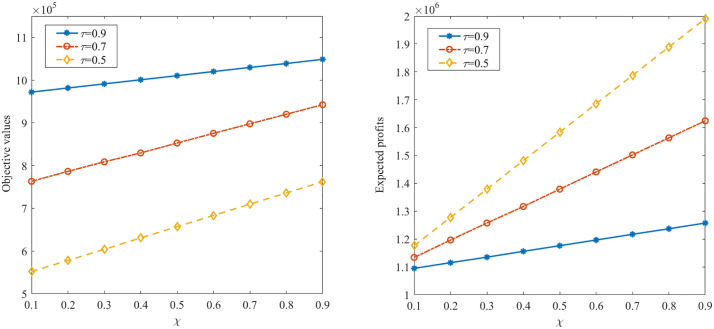
Objective values and expected profits with different χ and τ.

Table 5 further presents the objective values and expected profits under various risk aversion parameter χ and confidence level τ. As demonstrated in Table 5, the objective values and expected profits all increase as either the value of χ or τ increasing. Fig 2 further illustrates the variation of objective values and expected profits with the increasing of risk aversion parameter χ. This is because the decision-maker is more risk-averse with the values of τ decreasing, resulting in more conservative decisions. In addition, as risk aversion parameter χ increases, the decision-maker would place a higher value on the QD, resulting in a lower quantile deviation and a more concentrated distribution of profits.

**Table 5:** Objective values and expected profits with different χ and τ.

χ	τ=0.9		τ=0.7		τ=0.5	
	Obj	Exp	Obj	Exp	Obj	Exp
0.1	972,070	1,094,800	763,278	1,133,900	552,036	1,176,300
0.2	981,695	1,115,200	786,625	1,196,500	578,067	1,277,600
0.3	991,319	1,135,000	808,840	1,257,500	604,077	1,378,900
0.4	1,000,944	1,155,800	829,682	1,316,700	630,834	1,481,600
0.5	1,010,507	1,176,100	852,836	1,379,000	656,972	1,583,100
0.6	1,020,193	1,196,500	875,555	1,440,700	683,483	1,685,300
0.7	1,029,818	1,217,000	897,856	1,501,800	709,738	1,787,100
0.8	1,038,960	1,236,900	920,186	1,563,000	736,048	1,889,000
0.9	1,048,778	1,257,400	942,529	1,624,200	762,347	1,990,800

### 5.3. Performance evaluations

#### 5.3.1. Effects of μ in WDRJCCP model.

The Wasserstein ball’s radius μ serves as an input in the ambiguity set. We test the effects of μ on the out-of-sample performances for different sample sizes |N|={20,200}, and the values of μ∈{0,0.1,0.2,0.3,0.4,0.5}. It should be noted that as the number of sample sizes |N| increases, the confidence level of the true distribution ℙ within the ambiguity set rises accordingly. Consequently, the Wasserstein distance, which measures the distance between the empirical distribution and the actual distribution, will gradually decrease [45]. Two randomly generated instances are evaluated and the results are presented in Table 6. To assess the out-of-sample performance, many indicators are used, where Obj represents the objective value, Aver is the average out-of-sample profit, Std denotes the standard deviation of the out-of-sample profit, and Wor is the worst-case out-of-sample profit. These indicators are also adopted in the subsequent analyses. It can be seen from Table 6 that the performances are indeed influenced by the value of μ. Generally, the cases of |N|=200 perform better than those of |N|=20, which shows that increasing the sample size contributes to improving the quality of the solutions. The cases of μ=0.4 and 0.5 seem to perform better than other cases when |N|=20, whereas the cases of μ=0 and 0.1 outperform the others when |N|=200. Intuitively, a small sample size indicates little information about the true distribution, and a larger μ leads to more robust solutions that better hedge against ambiguity. Conversely, with a larger sample, more information can be extracted from the data, allowing for less conservative decisions with a smaller μ. Therefore, a larger (or smaller) μ should be chosen when the sample size is small (or large). When sufficient data is accessible, the SP model can be applied directly (i.e., μ=0). In contrast, considering the distributional ambiguity and utilizing the WDRJCCP model are particularly valuable when the available sample data is limited.

**Table 6:** Effects of μ on the performances.

Instance	μ	|N|=20				|N|=200			
		Obj	Aver	Std	Wor	Obj	Aver	Std	Wor
1	0	983,885	1,419,101	14,528	987,655	987,617	1,866,676	13,333	1,273,448
	0.1	983,881	1,419,105	14,523	987,660	987,613	1,866,673	13,325	1,274,658
	0.2	983,877	1,419,107	14,545	987,661	987,609	1,866,670	13,258	1,282,034
	0.3	983,873	1,419,110	14,542	987,675	987,605	1,866,671	13,217	1,293,792
	0.4	983,869	1,419,112	14,536	987,677	987,601	1,866,668	13,214	1,367,240
	0.5	983,865	1,419,113	14,580	987,664	987,597	1,866,668	13,225	1,365,432
2	0	996,540	1,551,168	14,895	1,002,500	1,001,250	1,956,028	13,552	1,225,800
	0.1	996,535	1,551,172	14,358	1,002,740	1,001,245	1,956,025	13,125	1,256,800
	0.2	996,527	1,551,174	14,652	1,004,520	1,001,237	1,956,022	13,114	1,285,630
	0.3	996,524	1,551,177	14,553	1,003,260	1,001,234	1,956,015	13,283	1,265,890
	0.4	996,520	1,551,179	14,621	1,004,210	1,001,230	1,956,007	13,321	1,269,470
	0.5	996,506	1,551,177	14,663	1,003,500	1,001,216	1,956,001	13,451	1,275,650

In the realm of omnichannel retailing, sales data are complex and diverse. Moreover, as market competition intensifies and customer preferences evolve rapidly, the relevance of historical data is decreasing as time passes. For instance, the sales data of a once-popular fashion item may lose its predictive value when new fashion trends emerge. Consequently, data insufficiency is a common issue in the practice of omnichannel retail management. Therefore, it is significant to consider distributional ambiguity and adopt appropriate optimization methods. When the sample size is limited, by taking distributional ambiguity into account, enterprises can obtain more robust decisions.

#### 5.3.2. Model comparison.

To assess the performance of the developed model, comparative analyses between the WDRJCCP and SP models are conducted. Specifically, the SAA method is applied to solve the SP model by adopting the empirical distribution to approximate the true distribution. The problem size and computational results of the WDRJCCP and SP models are summarized in Table 7. As shown in Table 7, the SP model contains fewer continuous variables and constraints compared to the WDRJCCP model. The reason is that auxiliary variables and constraints are introduced to obtain the tractable formulation in the WDRJCCP model. In addition, the SP model yields a higher objective value than the WDRJCCP model. This is because the SP approach maximizes the MQD based on a known empirical distribution, while the data-driven WDRJCCP approach aims for the maximization of the WMQD based on the true distribution.

**Table 7:** Comparisons of computational results of WDRJCCP model and SP model.

Model	Continuous variables	Constraints	Obj	Time(s)
WDRJCCP	6,908	4,188	1,014,100	9.21
SP	6,509	3,645	1,606,210	7.87

**Fig 3:**
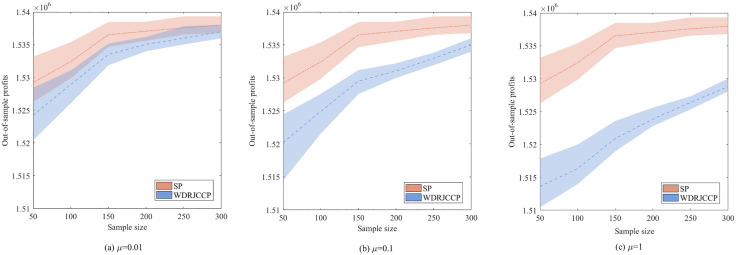
Out-of-Sample profits under different sample size.

**Fig 4:**
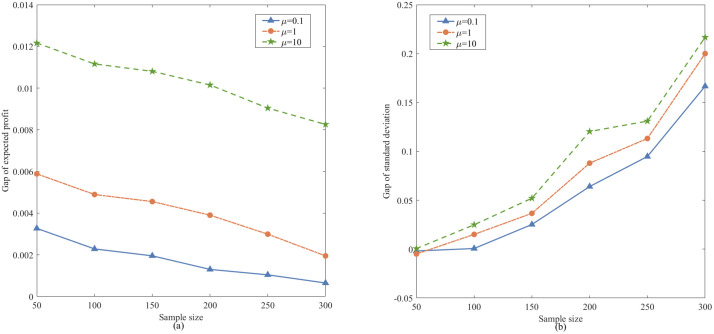
The gap of expected profit and standard deviation.

To further compare profit performance between SP and WDRJCCP models, a series instances are generated with increasing sample sizes, i.e., |N|={50,100,150,200,250,300}. Fig 3 shows the results with different values of μ, where the shaded area represents the 25% and 75% quantiles of the out-of-sample profit, and the line represents the mean of the out-of-sample profit. It can be observed from Fig 3 that the out-of-sample profits for both the SP and WDRJCCP models rise as the sample size grows. The reason for this phenomenon is that a larger sample size contributes to a more compact uncertainty set, and the demand information in the dataset will be more accurate. Fig 4 further illustrates the standard deviation and expected profit gaps between the SP and WDRJCCP models. With the sample size increasing, the expected profit gap decreases while the standard deviation gap increases. It further can be seen that the expected profits under the SP model would be no more than 1.2% higher than those under the WDRJCCP model. However, it is noteworthy that the standard deviation under the SP model is higher than that under the WDRJCCP model, with the potential to be over 20% higher, particularly when sample sizes are not small. The results indicate that the data-driven WDRJCCP model effectively mitigates the risks associated with uncertain demand at a relatively small cost with relatively larger sample sizes. Hence, the decision-makers are advised to meticulously maintain records of daily sales data, thereby enhancing the efficacy of decision-making processes.

**Fig 5:**
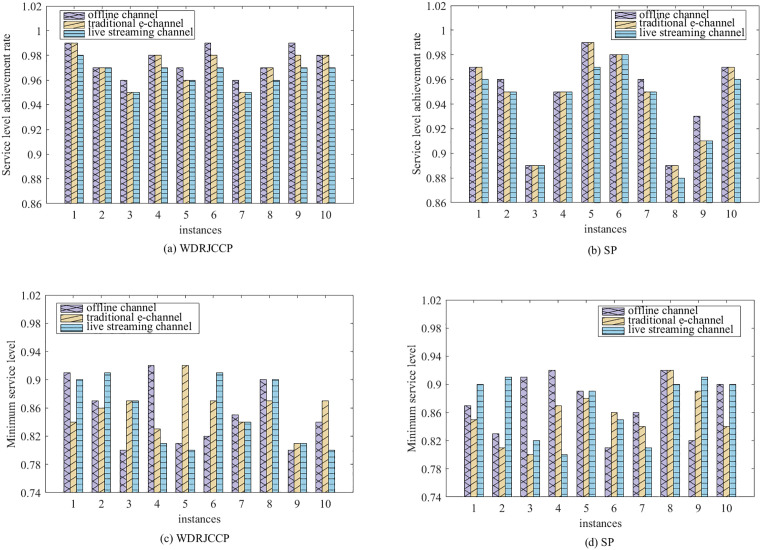
Service level under WDRJCCP and SP models.

Furthermore, ten instances with |N|=100 are randomly generated to discuss the service level achievement rate in different channels under SP and WDRJCCP models. As Fig 5 shows, regarding the SP model, the service level achievement rates exceed 0.95 except in instance 3 and 8 where it is below 0.9. Difference between the observed and true probability distribution prevents the SP model from guaranteeing that the violation probability remains within the specified levels. By contrast, the service level achievement rates obtained from the WDRJCCP model are consistently greater than 0.95. In addition, the difference between the SP and WDRJCCP models in terms of minimum service levels is not significant. Both the SP and the WDRJCCP models have a minimum service level of 0.8. Although the low service level may occur in WDRJCCP model, the consideration of chance constraints can effectively ensure that each channel has a high probability of meeting service level requirements, Therefore, the WDRJCCP model, which accounts for the worst-case scenario, is capable of managing uncertainty efficiently and ensuring more reliable service levels.

**Fig 6:**
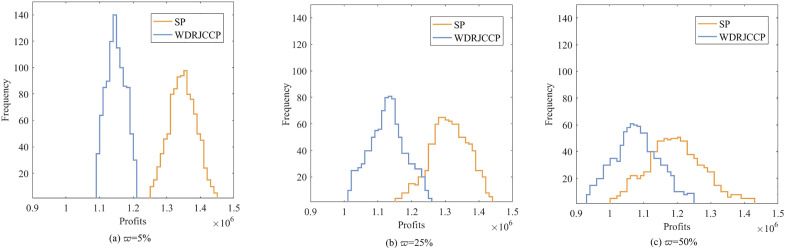
Out-of-sample profits under WDRJCCP and SP models with different $\begin{array}{c} \varpi \end{array}$.

A further comparison between WDRJCCP and SP models are presented. Similar to [48,54], we assume the uncertain demand $\xi_{j}$ follows a uniform distribution, denoted as $\xi \sim [\xi^{-},\xi^{+}]$. A perturbation parameter ϖ is introduced to describe the variation level, and a larger ϖ represents a higher variation level. Therefore, a parameterized uniform distribution $\left[(1-\varpi )\xi^{-},(1+\varpi )\xi^{+}\right]$ can be obtained, where ϖ takes values from the set {5%, 25%, 50%}. The instances with |N|=1000 are generated from this uniform distribution to test the performance of the optimal solutions obtained from the WDRJCCP and SP models. The histograms of out-of-sample profits are demonstrated in Fig 6, and Table 8 further presents the statistical measures for each histogram. As shown in Fig 6, there are distinct separation between the WDRJCCP and SP models when ϖ=5%, and the gap is gradually narrowing as ϖ increases. In addition, the profit distributions under the WDRJCCP model are more concentrated than those under the SP model for all the case of ϖ= 5%, 25%, and 50%. This finding demonstrates that the performance premium of the WDRJCCP model in handling uncertainty disturbances is more evident when the uncertainty perturbation is large. Furthermore, as indicated by the value of quantiles and Std in Table 8, the WDRJCCP model is more stable than the SP model. The above results reveal that our developed WDRJCCP model is more effective in the avoiding worst-case scenarios and producing more robust solutions, which is attractive to risk-averse decision-makers.

**Table 8:** Statistics of out-of-sample performances under WDRJCCP and SP models.

ϖ	Model	0.25-quantile	0.75-quantile	Aver	Std
5%	SP	1,297,200	1,325,200	1,345,200	15,022
	WDRJCCP	1,152,600	1,166,500	1,185,800	10,134
25%	SP	1,255,400	1,397,200	1,304,700	19,501
	WDRJCCP	1,135,600	1,206,300	1,173,500	12,352
50%	SP	1,175,200	1,425,200	1,244,100	22,966
	WDRJCCP	1,122,100	1,192,300	1,162,100	17,470

A comparison of the WDRJCCP models based on the WMCVaR and WMQD criterion is provided in Table 9. It can be observed that the objective values increase as either the value of χ or τ increases under both WMQD- and WMCVaR-based models. Additionally, the WMCVaR-based model consistently yields lower objective values compared to the WMQD-based model, no matter how the values of χ or τ are varied. Therefore, Obj.Gap are greater than 0, and it is increasing as the value of τ decreases. The results demonstrate that as the decision-maker is more risk-averse, the WMCVaR-based model will produce more conservative solutions compared to the WMQD-based model.

**Table 9:** Comparison of objective values under WDRJCCP models based on WMCVaR and WMQD.

χ	τ=0.9		Obj.Gap^a^ (%)	τ=0.5		Obj.Gap^a^(%)
	WCVaR	WMQD		WCVaR	WMQD	
0.1	1,038,555	1,038,559	0.0004	614,705	825,661	25.5500
0.2	1,142,410	1,142,419	0.0007	717,156	928,869	22.7926
0.3	1,246,266	1,246,278	0.0009	819,606	1,032,076	20.5867
0.4	1,350,122	1,350,138	0.0012	922,057	1,135,284	18.7818
0.5	1,453,978	1,453,997	0.0013	1,024,508	1,238,492	17.2778
0.6	1,557,833	1,557,857	0.0015	1,126,959	1,341,699	16.0051
0.7	1,661,689	1,661,716	0.0016	1,229,410	1,444,907	14.9143
0.8	1,765,544	1,765,575	0.0018	1,331,860	1,548,115	13.9689
0.9	1,869,400	1,869,435	0.0019	1,434,311	1,651,322	13.1417
1.0	1,973,255	1,973,294	0.0020	1,536,762	1,754,530	12.4118

^a^Obj.Gap represents the gap between the objective values of WMQD-based and WMCVaR-based models

### 5.4. Sensitivity analyses

To investigate the impact of model parameters on the optimal decisions, sensitivity analyses are conducted by varying the fulfillment cost, technical service fee, customer channel preference ratio, time-inconsistent preference, free-riding degree. All analyses are conducted under the case of |N|=100, χ=0.5 and τ=0.1.

#### 5.4.1. The analysis of fulfillment cost.

**Fig 7:**
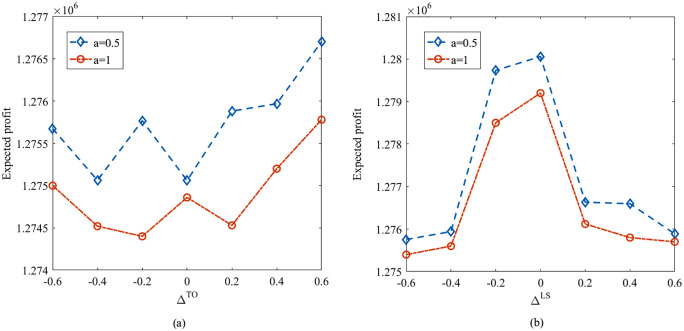
The expected profits with different fulfillment costs.

Next, we will examine how the unit fulfillment costs of traditional e-channel and live streaming channel affect the solution results. Note that the minimum unit fulfillment cost of traditional e-channel and live streaming channel are varied by ${\hat{\underset{―}{m}}}_{j^{\prime }j}^{o}={\underset{―}{m}}_{j^{\prime }j}^{o}+\Delta^{\text{TO}}$ and ${\hat{\underset{―}{m}}}_{j^{\prime }j}^{b}={\underset{―}{m}}_{j^{\prime }j}^{b}+\Delta^{\text{LS}}$, respectively, where ${\underset{―}{m}}_{j^{\prime }j}^{o}$ and ${\underset{―}{m}}_{j^{\prime }j}^{b}$ are randomly generated according to Table 3. $\Delta^{TO}$ and $\Delta^{LS}$ are changed from −0.6 to 0.6 in increments of 0.2. The results are illustrated in Fig 7. The counterintuitive phenomenon is that the expected profits do not always decrease as the unit fulfilment costs increase. As $\Delta^{TO}$ increases, the expected profits show a slight upward trend. In addition, as $\Delta^{LS}$ increases, the expected profits first increase and then decrease. The reason for this phenomenon is that as the unit fulfillment costs increase, the retailer can flexibly adjust pricing strategies and optimize cost structures to adapt to the increase in fulfillment costs, thereby potentially increasing profits in certain situations. Furthermore, with the marginal increase of the fulfillment cost increasing (i.e., a increasing), the expected profits decrease.

This finding can be further illustrated through real-world examples. Take fresh food e-commerce as an example, customers increasingly expect not only rapid delivery but also superior protective packaging to prevent damage in transit. Meeting these expectations inevitably leads to higher fulfillment costs. However, this cost pressure can catalyze strategic adaptation. A case in point is Freshippo, a mid-to-high-end fresh food e-commerce platform in China (https://www.freshippo.com/). To differentiate itself, Freshippo guarantees delivery within 30 minutes for online orders. While this rapid delivery service significantly increases its fulfillment expenses, it fundamentally enhances the shopping experience by offering unparalleled convenience and ensuring product freshness. Crucially, this superior service allows Freshippo to cultivate a brand image associated with premium quality and reliability. Enhanced brand perception and the resulting customer loyalty create significant scope for price increases. Customers who acknowledge and value the convenience and quality are willing to pay a higher price. Consequently, the increased revenue can offset the increased fulfillment costs, leading to higher overall profitability.

#### 5.4.2. The analysis of technical service fee.

**Fig 8:**
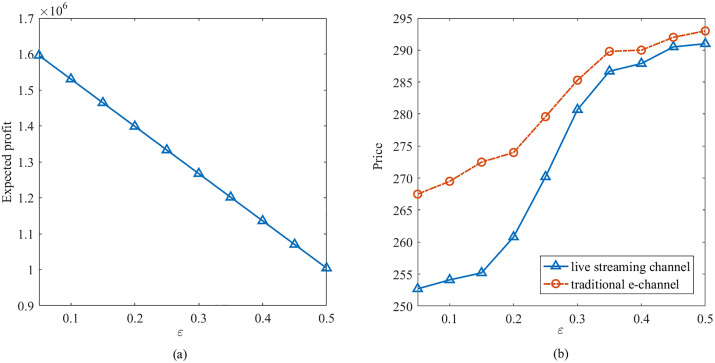
The decision results with different technical service fee.

Fig 8 demonstrates the effects of the technical service fee ε on the optimal decisions. The technical service fee ε is changed from 0.05 to 0.5 in increments of 0.05. It can be seen from Fig 8 that the expected profits decrease with the technical service fee ε increasing. Additionally, the product price is elevated in both the live streaming channel and the traditional e-channel. This is because as the technical service fee ε increases, the retailer will raise the product price to compensate for the profit loss. In order to reduce cannibalization between channels, the retailer simultaneously increases the product price in the traditional e-channel. Although the prices of the products in the live streaming channel and traditional e-channel rise, it does not contribute to the increase in profits.

#### 5.4.3. The analysis of customer channel preference ratio.

**Fig 9:**
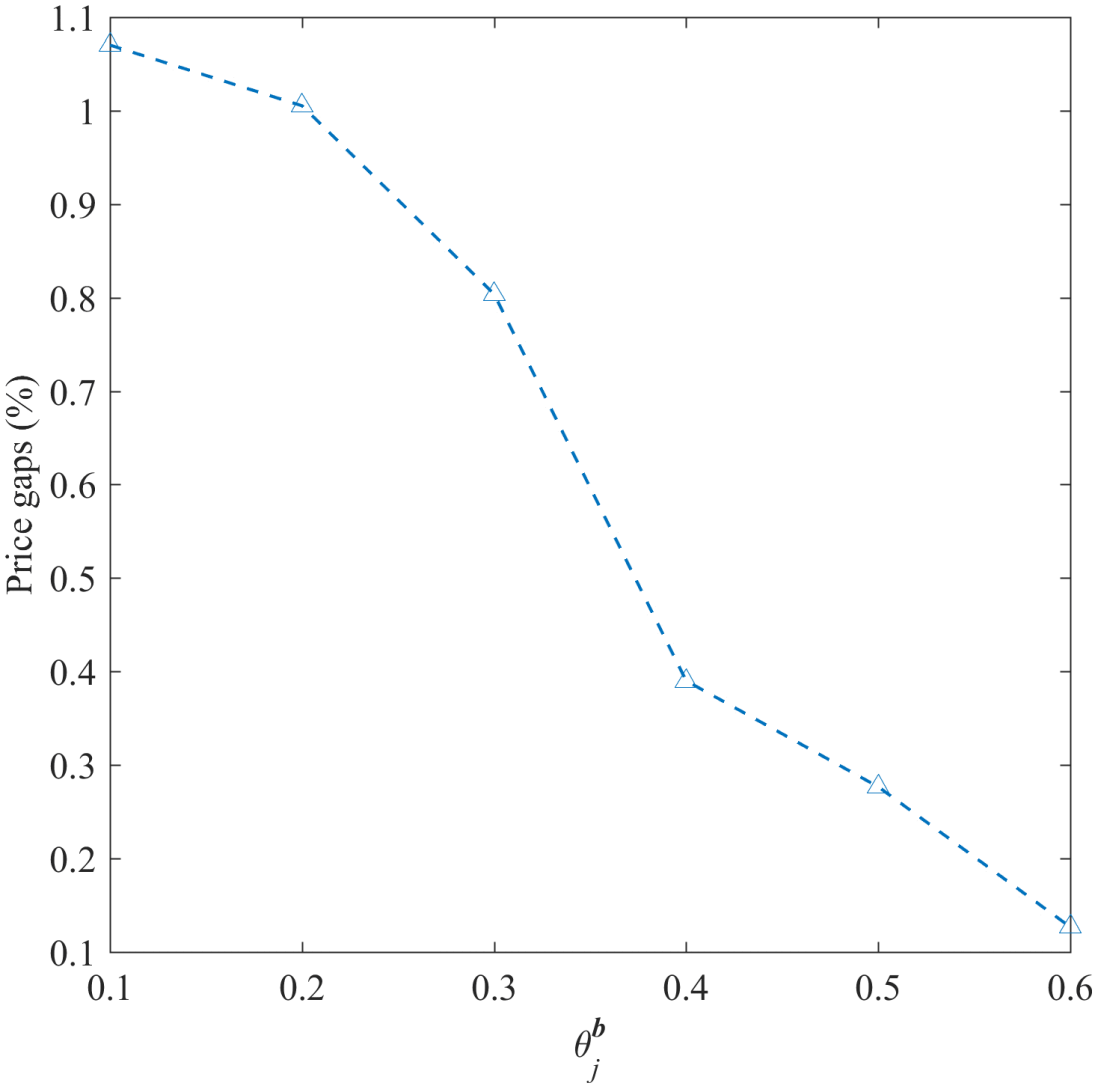
The price gap between live streaming channel and traditional e-channel.

We next investigate the impacts of customer channel preference ratio on the optimal decisions. The offline channel preference ratio $\theta_{j}^{f}$ is fixed at 0.3, and $\theta_{j}^{o}$ varies from 0.6 to 0.1 in steps of 0.1. Correspondingly, $\theta_{j}^{b}$ varies from 0.1 to 0.6 in steps of 0.1. The results are illustrated in Table 10 and Fig 9. It can be observed that as the live streaming channel preference ratio $\theta_{j}^{b}$ increases, the objective values decrease significantly, while the expected profits initially increase slightly then decrease evidently. In addition, the ordering quantity decreases while the replenishment quantity increases with the increase of $\theta_{j}^{b}$.

**Table 10:** Optimal solutions under different customer channel preference ratio.

[$\;\theta_{j}^{o}$, $\;\theta_{j}^{b}$]	Obj	Exp	Ordering Quantity	Replenishment quantity	Price
[0.6,0.1]	1,074,290	1,008,906	10,438	6,133	[299.02,299.02,295.82]
[0.5,0.2]	1,027,800	1,008,991	10,362	6,518	[299.52,299.52,296.01]
[0.4,0.3]	981,570	1,009,077	10,287	6,903	[299.82,299.82,297.41]
[0.3,0.4]	934,530	1,008,982	10,209	7,158	[299.98,299.98,298.81]
[0.2,0.5]	887,900	1,007,842	10,133	7,671	[300,300,299.17]
[0.1,0.6]	841,490	1,006,540	10,058	8,056	[300,300,299.62]

As the live streaming channel gains popularity, a growing number of customers are shifting to this channel. When the live streaming channel preference ratios are lower than traditional e-channel preference ratios, the retailer will benefit from the opening of the live streaming channel. However, as the number of customers in the live streaming channel increases, the traditional e-channel is being cannibalized, and the price in the live streaming channel tends to be lower, resulting in lower profitability. Additionally, flexibility in inventory allocation is a significant concern for the retailer under omnichannel retailing. As the live streaming channel preference ratio $\theta_{j}^{b}$ increases, the retailer must adjust inventory strategy. Satisfying the demands of live streaming channel is often the lowest priority, and the profits gained from live streaming channel may not be enough to offset the ordering and inventory holding costs. Therefore, as customer demands in live streaming channel increase, the retailer chooses to order fewer products to avoid operational losses. However, the replenishment quantity from the DC to stores has not decreased, indicating that the retailer will adopt more in-store inventory to satisfy online demand, as the fulfillment cost of stores is lower than that of the DC. Moreover, as observed from Fig 9, the profit gaps, expressed as (price in offline channel/traditional e-channel – price in live streaming channel)/ price in offline channel/traditional e-channel, become narrower with the increase of $\;\theta_{j}^{b}$. Since customers are sensitive to the price, the retailer will narrow the price gaps between different channels to avoid more customers shifting to the live streaming channel. Additionally, the retailer also expects to make up for the decline in profitability of the live streaming channel by raising prices in other channels.

#### 5.4.4. The analysis of time-inconsistent preference.

**Fig 10:**
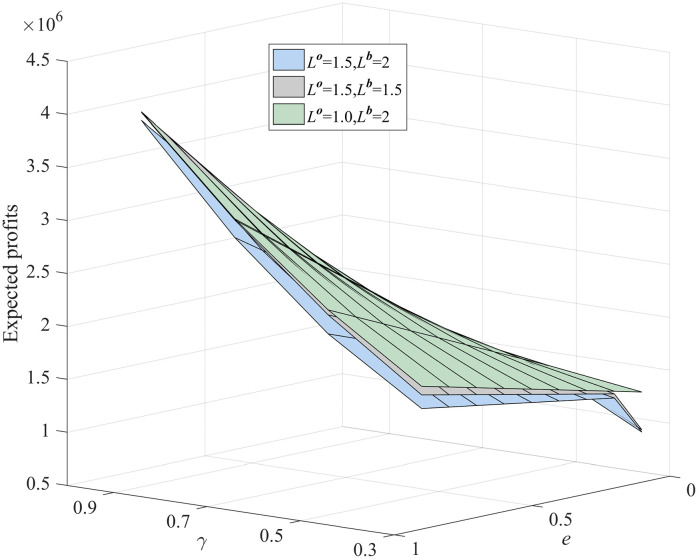
Expected profit under different combinations of e and γ.

**Fig 11:**
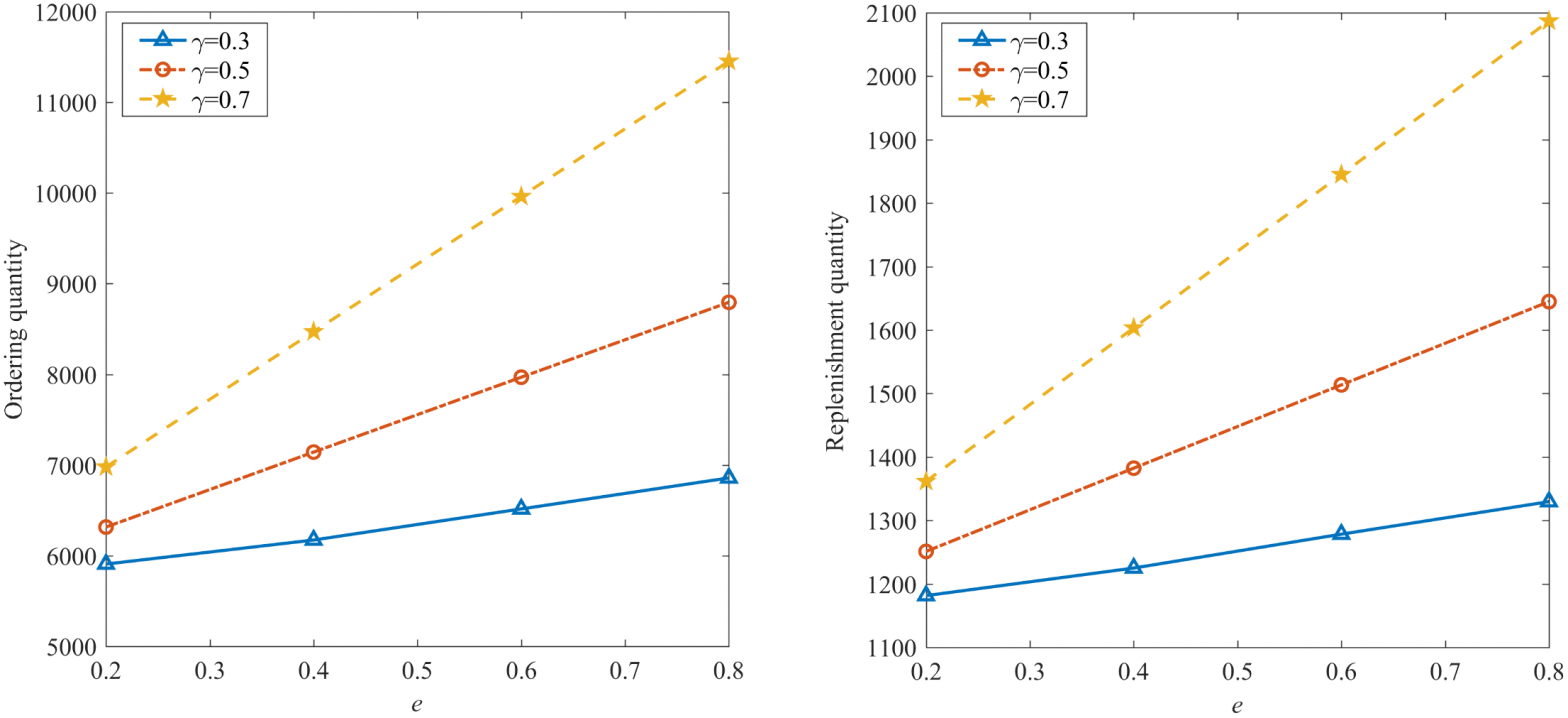
The impacts of e and γ on ordering quantity and replenishment quantity.

To investigate the effects of time-inconsistent preference, the numerical experiments are performed under different short-term and long-term factors with different delivery time. When customers show time-inconsistent preferences, the changes in short-term factor e and long-term factor γ can affect customer demand, which in turn affect the retailer’s profitability. The short-term factor e varies from 0.1 to 0.9 in steps of 0.1, and long-term factor γ varies from 0.3 to 0.9 in steps of 0.2. Fig 10 illustrates the trend of expected profits under different delivery times with the changes of e and γ. As e (γ) increases, the expected profits show a rising trend, which is increasingly evident with the impact of γ (e). Additionally, shortening the delivery time, either in the traditional e-channel or in the live streaming channel, can lead to increased profits. Fig 11 further shows the effects of e and γ on the inventory decisions. As e (γ) increases, the ordering quantity significantly increases and the upward trends become increasingly evident with the impact of γ (e).

#### 5.4.5. The analysis of free-riding behavior.

**Fig 12:**
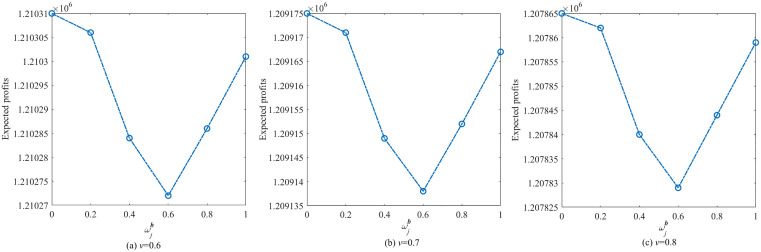
Effects of $\omega_{j}^{b}$ on the expected profits under different live streaming selling efforts.

We next explore the impacts of free-riding behavior on the optimal decisions. We vary free-riding degree $\omega_{j}^{b}$ from 0 to 1 with step length 0.2. Assuming the values of $\omega_{j}^{f}$ and $\omega_{j}^{o}$ are equal. Table 11 and Fig 12 demonstrate the effects of $\omega_{j}^{b}$ on expected profits under different LSS efforts. It is evident that that the expected profits decrease first and then increase as the value of $\omega_{j}^{b}$ increases. A larger value of $\omega_{j}^{b}$ represents a smaller percentage of free-riding customers, who are attracted to the market by the LSS, purchase products from offline channel or traditional e-channel. When $\omega_{j}^{b}$ is below a certain threshold, the positive spillover effect of live streaming selling is significant. The price in the live streaming channel is reduced to attract more customers. However, the positive impacts on profit from increased demand in offline channel or traditional e-channel are more pronounced. Therefore, when $\omega_{j}^{b}$ is less than a certain threshold, the expected profits decrease as $\omega_{j}^{b}$ increases. Additionally, when $\omega_{j}^{b}$ exceeds a certain threshold, an increase in $\omega_{j}^{b}$ leads to a rise in demand for the live streaming channel. Consequently, the product prices in the live streaming channel increase. Although this comes at the expense of losing customers who are attracted to the live streaming channel by its low prices, the overall increase in demand in the live streaming channel, as well as the increase in the price, can still improve the retailer’s profit. Furthermore, as the level of LSS effort increases, the expected profits decrease. A higher level of LSS efforts can enhance the free-riding effects, yet the retailer’s profit may not necessarily better off. This suggests that the more demand resulting from increased sales efforts of live streaming does not offset the increased cost, ultimately harming the retailer’s profit.

**Table 11:** Effects of $\omega_{j}^{b}$ on the expected profits under different live streaming selling efforts.

$\omega_{j}^{b}$	v=0.6		v=0.7		v=0.8	
	Exp	Price	Exp	Price	Exp	Price
0	1,210,310	[299.8,299.8,299.6]	1,209,175	[299.4,299.4,298.9]	1,207,865	[299.8,299.8,298.4]
0.2	1,210,306	[299.7,299.7,299.0]	1,209,171	[299.7,299.7,298.6]	1,207,862	[299.9,299.9,298.2]
0.4	1,210,284	[299.6,299.6,298.9]	1,209,149	[299.6,299.6,298.6]	1,207,840	[299.6,299.6,298.0]
0.6	1,210,272	[299.9,299.9,298.6]	1,209,138	[299.7,299.7,298.2]	1,207,829	[299,6,299.6,297.8]
0.8	1,210,286	[299.6,299.6,297.9]	1,209,152	[299.5,299.5,297.8]	1,207,844	[299.8,299.8,297.5]
1.0	1,210,301	[299.8,299.8,297.4]	1,209,167	[299.4,299.4,297.2]	1,207,859	[299.3,299.3,297.1]

### 5.5. Managerial insights

According to the proposed model and experimental results, several important managerial insights are presented in the following.

(1) Customer demand in omnichannel retailing is often uncertain, and various risks associated with this cannot be ignored. Moreover, the exact probability distribution information is unavailable in advance, ignoring it will lead to a weak decision. The comparative results of the out-of-sample performance indicate that the proposed WDRJCCP model outperforms the SP model in terms of hedging against the risks of inaccurate demand estimation. In addition, a larger dataset benefits the profits and helps to guarantee the reliability of the decision-making when accurate probability distribution information is unavailable. Therefore, keeping good records of sales data and adopting data-driven decision-making models for managing real-world omnichannel operations are significantly important. However, sales data are always complex and diverse, and data insufficiency is a common issue in the practice of omnichannel retail management. Hence, it is essential to consider distributional ambiguity of uncertain parameters and adopt appropriate optimization methods.
(2) Smaller violation probability values indicate higher customer demand satisfaction. The consideration of chance constraints can effectively ensure that each channel has a high probability of meeting service level requirements. The WDRJCCP model, which accounts for the worst-case scenario, is capable of effectively managing uncertainty, thereby ensuring more robust service levels. This not only increases customer satisfaction but also builds trust and loyalty, which are crucial for long-term customer retention and repeat business. In addition, this proactive risk management helps in minimizing disruptions and ensuring consistent service level, even under adverse conditions. Therefore, the decision-maker can use this approach to optimize resource allocation and reasonably arrange the operations of various channels, thereby improving overall service levels and customer satisfactions.
(3) Through the analysis of fulfillment costs of online orders, it can be found that the expected profits do not always decrease as the unit fulfilment costs increase. This suggests that retailers should not solely focus on reducing fulfillment costs. Instead, they should adopt flexible pricing strategies to maintain profit levels even when facing higher fulfillment costs. In addition, the retailer is not advised to reduce fulfillment costs by investing in technology or increasing delivery time, as these actions may not always yield positive returns. However, they can conduct regular cost-benefit analyses to ensure that any investments in technology or changes in delivery times are justified and contribute to overall profitability.
(4) It can be found that the introduction of live streaming channel is not always profitable for retailer, and an increasing number of customers shifting to live streaming channel does not necessarily yield higher profits. The retailer need monitor and analyze customer channel preferences continuously. It is essential for the retailer to appropriately control the live streaming channel preference and reasonably adjust inventory allocation and pricing strategies to avoid customer loss and profit decline due to changes in channel preferences.
(5) Through the analysis of customers’ time-inconsistent preferences, it becomes evident that the short-term and long-term factors can affect retailer’s profitability and ordering decisions. Therefore, the retailer should have a precise understanding of customers’ inconsistent preferences regarding delivery time, which is conducive to accurately grasping the market changes and making more effective operational decisions. Additionally, the expected profits will increase as the delivery times of either the traditional e-channel or live streaming channel are shortened. Hence, it is essential for the retailer to value the construction of the logistics and distribution system to shorten the delivery time and improve customer satisfaction.
(6) From the analysis of free-riding behavior, it is evident that free-riding behavior has significant impacts on the retailer’s profits. First, when the free-riding degree $\omega_{j}^{b}$ is less than a certain threshold, the retailer should reasonably control the value of $\omega_{j}^{b}$. The retailer can adopt some restrictive measures, such as live streaming exclusive coupons and membership system for live streaming, to restrict the free-riding customers to make purchases from other channels and maintain a high level of profits. However, when the free-riding degree $\omega_{j}^{b}$ exceeds a certain threshold, the retailer can flexibly adjust pricing strategy to capitalize on free-riding behavior, thereby promoting profit growth. Furthermore, while increasing the level of LSS effort can enhance free-riding effect, the retailer’s profits will not necessarily become better off. This implies that the retailer should balance the investment and benefit in live streaming channel and determine the appropriate level of LSS effort based on market size and customer behavior.

## 6. Conclusions

This article investigates the omnichannel pricing and inventory strategies considering LSS with uncertain demand and service level requirement. In addition to price sensitivity, customers are also sensitive to the delivery time and reveal time-inconsistent preferences, which is modelled by using hyperbolic discounting. Furthermore, LSS can influence demand and benefit for other channels to take free-riding. A data-driven Wasserstein DRJCCP model based on WMQD criterion is proposed to determine the robust ordering quantity, replenishment quantity, order fulfillment, and prices of product in different channels. By adopting the Wasserstein metric, the data-driven ambiguity set is designed. Leveraging the dual theory, CVaR approximation and linearization techniques, equivalent tractable formulations are obtained. Finally, we conduct the numerical experiments to illustrate the validity and application of the developed model. The computational results indicate that our developed model not only has superior performance in terms of hedging against uncertainty, but also provides powerful decision support for the omnichannel operations incorporating LSS.

There are other research directions that warrant further investigation. First, this study is conducted from the perspective of the monopolistic retailer. However, there are always competitors in real-world markets. Therefore, a pivotal direction for future research is to model the strategic interactions among multiple retailers using non-cooperative game theory and explore the market equilibrium. Furthermore, the level of LSS effort in the live streaming channel is assumed to be an exogenous variable in this study. Future research will formally model the effort level as a decision variable within a game-theoretic framework, investigating the optimal selling format and coordination mechanisms between the retailer and the streamer. In addition, one of the most imperative directions for future work is the empirical validation of the framework on real-world sales datasets to further validate its practical efficacy. Moreover, a promising avenue for future research is to extend this framework to multi-period, multi-product model and investigate the dynamic pricing and inventory allocation problem. Developing effective algorithms leveraging decomposition techniques, such as Benders decomposition or column generation to solve the multi-period, multi-product model, represents a valuable approach to enhancing computational efficiency in the future research.

## Supporting information

S1 Appendix The appendix provides all proofs of propositions. (DOC)
