# Supplementary material for: Omnichannel pricing and inventory strategies considering live streaming selling: A data-driven distributionally robust optimization approach
Source: PLoS One. 2026 Jan 7;21(1):e0338918. doi: 10.1371/journal.pone.0338918 (PMC12779160; doi:10.1371/journal.pone.0338918)
Supplement: S1 Appendix — (DOC) [file pone.0338918.s001.doc]

**Appendix**

**Proof of Proposition 1**

The minimization problem of  can be viewed as the maximization problem . According to the Wasserstein ambiguity set, if , then there is a joint distribution $\Pi$ such that , with

where represents the conditional distribution of $\xi$ given that $\hat{\xi}=\hat{\xi}_{i}$. Therefore, the maximization problem can be reformulated as

Since problem has at least one feasible solution under , $\forall i\in N$, and the third constraint in model are not binding, the Slater’s condition holds. Additionally, problem is bounded, which indicates that the strong duality holds. Therefore, the Lagrange function of problem are presented as follows

where $\eta_{i}$, $\pi$ are dual variables. The dual problem then is . If , then is unbounded, which results to a contradiction to the strong duality of problem . Therefore, holds. In this case,

with optimal solutions satisfying

Then, the dual formulation can be reformulated as follows:

It is obvious that the optimal solution $\eta_{i}$ should satisfy . And thus, the problem is equivalent to

Introducing auxiliary variables $\vartheta_{i}$, we can obtain the following formulations

$$\pi\geq0.$$

The left-hand side of can be converted into

where represents the dual norm, $\upsilon_{s}$ is the introduced variable. We adopt $l_{1}$-norm in this paper due to its computational benefits in DRO. The first equality is based on the definition of the dual norm, and the last equality can be given by the minmax theorem. Let $\Delta=\sum_{g\in G} \left(s^{g}\right)^{T}Y^{g}-\sum_{g\in G\backslash\{b\}} \left(p^{g}\right)^{T}S^{g}-\left(1-\varepsilon\right)\left(p^{b}\right)^{T}S^{b}+h^{T}\sigma+c^{T}I+\sum_{j\in J} \left(m_{j}^{o}\right)^{T}S_{j}^{o}+\sum_{j\in J} \left(m_{j}^{b}\right)^{T}S_{j}^{b}-V$， $Y^{f}=-A^{f}p+Bp^{b}+\Omega^{f}\lambda v-S^{f}$, $Y^{o}=-A^{o}p+Bp^{b}+\Omega^{o}\lambda v-S^{o}$, $Y^{b}=-A^{b}p^{b}+Bp+\Omega^{b}\lambda v-S^{b}$, $V=-h_{0}\sigma_{0}-wQ-nv^{2}$. Based on , problem - can be further transformed into

$$\begin{matrix} \min_{\pi,\tau_{i},\upsilon_{i}} \pi\mu+\frac{1}{\left|N\right|}\sum_{i\in N} \vartheta_{i} \\ \text{s.t.}\mathop{ \max}\limits_{\xi\in\Xi} \left\{\Delta+\sum_{g\in G} \left(s^{g}\right)^{T}\Theta^{g}\xi-\left(\upsilon_{i}\right)^{T}\left(\xi-\hat{\xi}_{i}\right)\right\}\leq\vartheta_{i},\forall i\in N, \\ \left\|\upsilon_{i}\right\|_{*}\leq\pi,\forall i\in N. \end{matrix}$$

Combining the support set $\Xi$ and dual transformations, $\max_{\xi\in\Xi} \left\{\Delta+\sum_{g\in G} \left(s^{g}\right)^{T}\Theta^{g}\xi-\left(\upsilon_{i}\right)^{T}\left(\xi-\hat{\xi}_{i}\right)\right\}$ is equivalent to the following problem

$$\begin{matrix} \min_{\varsigma_{i},\psi_{i}} \Delta+\sum_{g\in G} \left(s^{g}\right)^{T}\Theta^{g}\hat{\xi}_{i}+\left(\bar{\xi}-\hat{\xi}_{i}\right)^{T}\varsigma_{i}-\left(\underline{\xi}-\hat{\xi}_{i}\right)^{T}\psi_{i} \\ \text{s.t.} \left\|\sum_{g\in G} \Theta^{g}s^{g}-\varsigma_{i}+\psi_{i}\right\|_{*}\leq\pi,\forall i\in N, \\ \varsigma_{i}\geq0,\psi_{i}\geq0,\forall i\in N, \end{matrix}$$

where $\varsigma_{i}$, $\psi_{i}$ are the dual variables. And therefore, can be converted into

$$\begin{matrix} \min_{\pi,\vartheta_{i},\varsigma_{i},\psi_{i}} \pi\mu+\frac{1}{\left|N\right|}\sum_{i\in N} \vartheta_{i} \\ \text{s.t.} \Delta+\sum_{g\in G} \left(s^{g}\right)^{T}\Theta^{g}\hat{\xi}_{i}+\left(\bar{\xi}-\hat{\xi}_{i}\right)^{T}\varsigma_{i}-\left(\underline{\xi}-\hat{\xi}_{i}\right)^{T}\psi_{i}\leq\vartheta_{i},\forall i\in N, \\ \left\|\sum_{g\in G} \Theta^{g}s^{g}-\varsigma_{i}+\psi_{i}\right\|_{*}\leq\pi,\forall i\in N, \\ \varsigma_{i}\geq0,\psi_{i}\geq0,\forall i\in N. \end{matrix}$$

**Proof of** **Proposition 2**

The proof process is similar to the proof of proposition 1. First, we can transfer into the following formulation

where

For easy clarification, we can set $\Delta'_{1}=\left(1-\chi\tau+\chi\right)\Delta+\chi\delta$, $\Delta'_{2}=\left(1-\chi\tau\right)\Delta$, , . Therefore, can be converted into

Let , and according to , the minimization problem can be viewed as the maximization problem , which can be reformulated as

And the Lagrange function of problem are presented as follows

where $\eta'_{i}$, $\pi'$ are dual variables. The dual problem then is . If , then is unbounded, which results to a contradiction to the strong duality of problem . Therefore, $-\Lambda-\left|N\right|\eta'_{i}-\pi'\left\|\xi-\hat{\xi}_{i}\right\|\leq0$ holds. In this case,

with optimal solutions satisfying

Then, the dual formulation can be reformulated as follows:

$$\begin{matrix} \min_{\eta_{i},\pi\geq0} \pi'\mu+\sum_{i\in N} \eta'_{i} \\ \text{s.t.} -\Lambda-\left|N\right|\eta'_{i}-\pi'\left\|\xi-\hat{\xi}_{i}\right\|\leq0,\forall i\in N. \end{matrix}$$

It is obvious that the optimal solution $\eta'_{i}$ should satisfy . And thus, the problem is equivalent to

$$\min_{\eta'_{i},\pi'\geq0} \left\{\frac{1}{\left|N\right|}\sum_{i\in N} \max_{\xi\in\Xi} \left\{-\Lambda-\pi'\left\|\xi-\hat{\xi}_{i}\right\|\right\}+\pi'\mu\right\}.$$

Introducing auxiliary variables $\vartheta'_{i}$, we can obtain the following formulations

$$\text{s.t.} \max_{\xi\in\Xi} \left\{-\Lambda-\pi'\left\|\xi-\hat{\xi}_{i}\right\|\right\}\leq\vartheta'_{i},\forall i\in N,$$

$$\pi'\geq0.$$

The left-hand side of can be converted into

$$\begin{matrix} \max_{\xi\in\Xi} \left\{-\Lambda-\pi'\left\|\xi-\hat{\xi}_{i}\right\|\right\} \\ =\max_{\xi\in\Xi} \left\{-\Lambda-\max_{\left\|\upsilon_{i}\right\|_{*}\leq\pi} \left(\upsilon'_{i}\right)^{T}\left(\xi-\hat{\xi}_{i}\right)\right\} \\ =\min_{\left\|\upsilon_{i}\right\|_{*}\leq\pi} \max_{\xi\in\Xi} \left\{-\Lambda-\left(\upsilon'_{i}\right)^{T}\left(\xi-\hat{\xi}_{i}\right)\right\}, \end{matrix}$$

where represents the dual norm, $\upsilon'_{i}$ is the introduced variable. The first equality is based on the definition of the dual norm, and the last equality can be given by the minmax theorem. Based on , problem - can be further transformed into

$$\begin{matrix} \min_{\pi',\vartheta'_{i},\upsilon'_{i}} \pi'\mu+\frac{1}{\left|N\right|}\sum_{i\in N} \vartheta'_{i} \\ \text{s.t.}\mathop{ \max}\limits_{\xi\in\Xi} \left\{-\Lambda-\left(\upsilon'_{i}\right)^{T}\left(\xi-\hat{\xi}_{i}\right)\right\}\leq\vartheta'_{i},\forall i\in N, \\ \left\|\upsilon'_{i}\right\|_{*}\leq\pi',\forall i\in N. \end{matrix}$$

Combining the support set $\Xi$ and dual transformations, $\max_{\xi\in\Xi}\left\{-\Lambda-\left(\upsilon'_{i}\right)^{T}\left(\xi-\hat{\xi}_{i}\right)\right\}$ is equivalent to the following problem

where $\varsigma'_{i}$, $\psi'_{i}$ are the dual variables with respect to $\underline{\xi}\leq\xi\leq\bar{\xi}$. And therefore, can be converted into

Since , and we adopt $l_{1}$-norm in this paper, can be reformulated as

**Proof of** **Proposition 3**

According to the strong duality theorem,

Therefore, by introducing the auxiliary variables $t_{i}^{g}$ for $\forall i\in N$, is equivalent to

The constraint can be equivalently denoted aswhere and $\Xi_{2}=\Xi\backslash\Xi_{1}$. If $\hat{\xi}_{i}\in\Xi_{1}$, then $\max_{\xi\in\Xi_{2}} -\zeta^{g}\rho\left(\xi,\hat{\xi}_{i}\right)<0$ and hold. If $\hat{\xi}_{i}\in\Xi_{2}$, then $\max_{\xi_{i}\in\Xi_{2}} -\zeta^{g}\rho\left(\xi,\hat{\xi}_{i}\right)=0$ and hold. Under these results, we can obtain the following constraint

Specifically,

Furthermore, based on the definition of the norm and the duality theory, can be equivalently converted to , where $k_{ij}^{g}$ and $u_{ij}^{g}$ are the dual variables. Therefore, constraint can be transformed into the following formulation

Combining and , the CVaR approximation of the DRJCC can be represented by
